# Transient Response of Basal Ganglia Network in Healthy and Low-Dopamine State

**DOI:** 10.1523/ENEURO.0376-21.2022

**Published:** 2022-03-17

**Authors:** Kingshuk Chakravarty, Sangheeta Roy, Aniruddha Sinha, Atsushi Nambu, Satomi Chiken, Jeanette Hellgren Kotaleski, Arvind Kumar

**Affiliations:** 1 TCS Research, Tata Consultancy Services, Kolkata, 700160, India; 2 Division of System Neurophysiology, National Institute for Physiological Sciences, Okazaki, 444-8585, Japan; 3 Department of Physiological Sciences, SOKENDAI (Graduate University for Advanced Studies), Okazaki, 444-8585, Japan; 4 Department of Computational Science and Technology, School of Computer Science and Communication, KTH Royal Institute of Technology, Stockholm, SE-10044, Sweden; 5 Department of Neuroscience, Karolinska Institute, Stockholm, SE 171 77, Sweden

**Keywords:** basal ganglia, direct pathway, indirect pathway, network model, Parkinson’s disease, transient response

## Abstract

The basal ganglia (BG) are crucial for a variety of motor and cognitive functions. Changes induced by persistent low-dopamine (e.g., in Parkinson’s disease; PD) result in aberrant changes in steady-state population activity (β band oscillations) and the transient response of the BG. Typically, a brief cortical stimulation results in a triphasic response in the substantia nigra pars reticulata (SNr; an output of the BG). The properties of the triphasic responses are shaped by dopamine levels. While mechanisms underlying aberrant steady state activity are well studied, it is still unclear which BG interactions are crucial for the aberrant transient responses in the BG. Moreover, it is also unclear whether mechanisms underlying the aberrant changes in steady-state activity and transient response are the same. Here, we used numerical simulations of a network model of BG to identify the key factors that determine the shape of the transient responses. We show that an aberrant transient response of the SNr in the low-dopamine state involves changes in the direct pathway and the recurrent interactions within the globus pallidus externa (GPe) and between GPe and subthalamic nucleus (STN). However, the connections from D2-type spiny projection neurons (D2-SPN) to GPe are most crucial in shaping the transient response and by restoring them to their healthy level, we could restore the shape of transient response even in low-dopamine state. Finally, we show that the changes in BG that result in aberrant transient response are also sufficient to generate pathologic oscillatory activity in the steady state.

## Significance Statement

To understand how changes induced by low-dopamine (e.g., in Parkinson’s disease; PD) affect basal ganglia (BG) function, we need to identify the factors that determine the shape of BG responses to brief cortical stimuli. We show that the transient response of the BG is also affected by recurrent interactions within the subnuclei of the BG, and not just feedforward pathways. We found that input and local connectivity within the globus pallidus externa (GPe) are crucial for shaping the transient response. We also show that the same network changes may underlie both pathologic β band oscillations and aberrant transient responses. Our results highlight the importance of the recurrent connectivity within the BG and provide a coherent view of emergence of pathologic activity in PD.

## Introduction

Parkinson’s disease (PD) is a debilitating neurodegenerative brain disease with multiple cognitive and motor symptoms. Etiologically the disease is attributed to the progressive loss of dopaminergic neurons in the substantia nigra pars compacta. Dopamine affects neuronal excitability, synaptic strength and synaptic plasticity. Consistent with this, data from human patients and animal models show that persistent dopamine deficit results in a number of changes in the neuronal activity especially in the basal ganglia (BG). At the level of neuronal activity, in PD, synchronized β band oscillations (15–30 Hz) in the globus pallidus externa (GPe) and subthalamic nucleus (STN; Raz et al., 2000; Brown et al., 2001; Mallet et al., 2008; Tinkhauser et al., 2017) emerge along with an increase in spike bursts (Tachibana et al., 2011; Nambu et al., 2015). Recent experimental studies also pointed out the role of GPe subpopulation (arkypallidal: GPe-TA, and prototypical: GPe-TI) in orchestrating the oscillatory activity in the BG subnuclei (de la Crompe et al., 2020). In the striatum, firing rate of D2-type dopamine receptors expressing spiny projection neurons (D2-SPN) is increased whereas firing rate of D1-SPNs is reduced (Mallet et al., 2006; Sharott et al., 2017) in the PD conditions. Moreover, while cortical inputs to D2-SPN are enhanced, inputs to D1-SPN are weakened (Parker et al., 2016; Ketzef et al., 2017; Filipović et al., 2019). The aforementioned changes in the activity and structure of the BG are persistent and indicate a change in “operating point” of the BG. But these observations do not provide mechanistic links between behavior deficits of PD and BG activity.

During action-selection or decision-making tasks the BG receives transient inputs (Gage et al., 2010) from different cortical regions. It is therefore important to understand how the response of the BG network to a transient cortical input is altered during PD condition. In a healthy state, transient cortical stimulation elicits predominantly a triphasic response (composed of early excitation, inhibition, and late excitation) in most neurons of the BG output nuclei, i.e., globus pallidus interna (GPi) or substantia nigra pars reticulata (SNr; Chiken and Nambu, 2013; Sano et al., 2013; Ozaki et al., 2017). The triphasic response is consistent with the predictions of a simple feedforward model of the BG involving the so-called direct, indirect and hyperdirect pathway (Albin et al., 1989; Jaeger and Kita, 2011). However, a small fraction of neurons in SNr (Sano and Nambu, 2019) or GPi (Iwamuro et al., 2017) can also show biphasic or monophasic responses. In low-dopamine conditions, the fraction of neurons showing triphasic, biphasic and monophasic responses is changed resulting in an altered population response.

To identify what determines the shape of BG transient responses we used a BG network developed by Lindahl and Kotaleski (2016). We found that, consistent with experimental data (Sano and Nambu, 2019) and the feedforward model of the BG (Albin et al., 1989), in healthy state, the SNr showed triphasic responses for brief cortical inputs at the population level. In the low-dopamine state, with the default settings, the SNr transient response was biphasic. However, by changing the strength of synapses along the direct (D1-SPN →SNr) and indirect pathways (D2-SPN →GPe-TI, and GPe-TI →STN) it was possible to observe the triphasic responses even in low-dopamine state. Interestingly, we found that changes in the transient response properties in PD state involve not only changes in the feed-forward connections (e.g., D1-SPN →SNr) but also recurrent interactions within BG subnuclei, e.g., the recurrent connections within the GPe (GPe-TA ↔GPe-TI) and between GPe and STN (GPe ↔STN). Next, we show that by restoring the connection from D2-SPN to GPe (D2-SPN →GPe-TI) to a normal value, even in low-dopamine state we can recover a transient response similar to that observed in healthy/normal state. Thus, the D2-SPN →GPe-TI emerged as the most important descriptor of the aberrant transient response. Interestingly, the same connections can also unleash β band oscillations (Kumar et al., 2011; Mirzaei et al., 2017). That is, the same changes underlie both the emergence of pathologic β band oscillations, and pathologic transient response.

## Materials and Methods

### Neuron model

**Figure 1.**
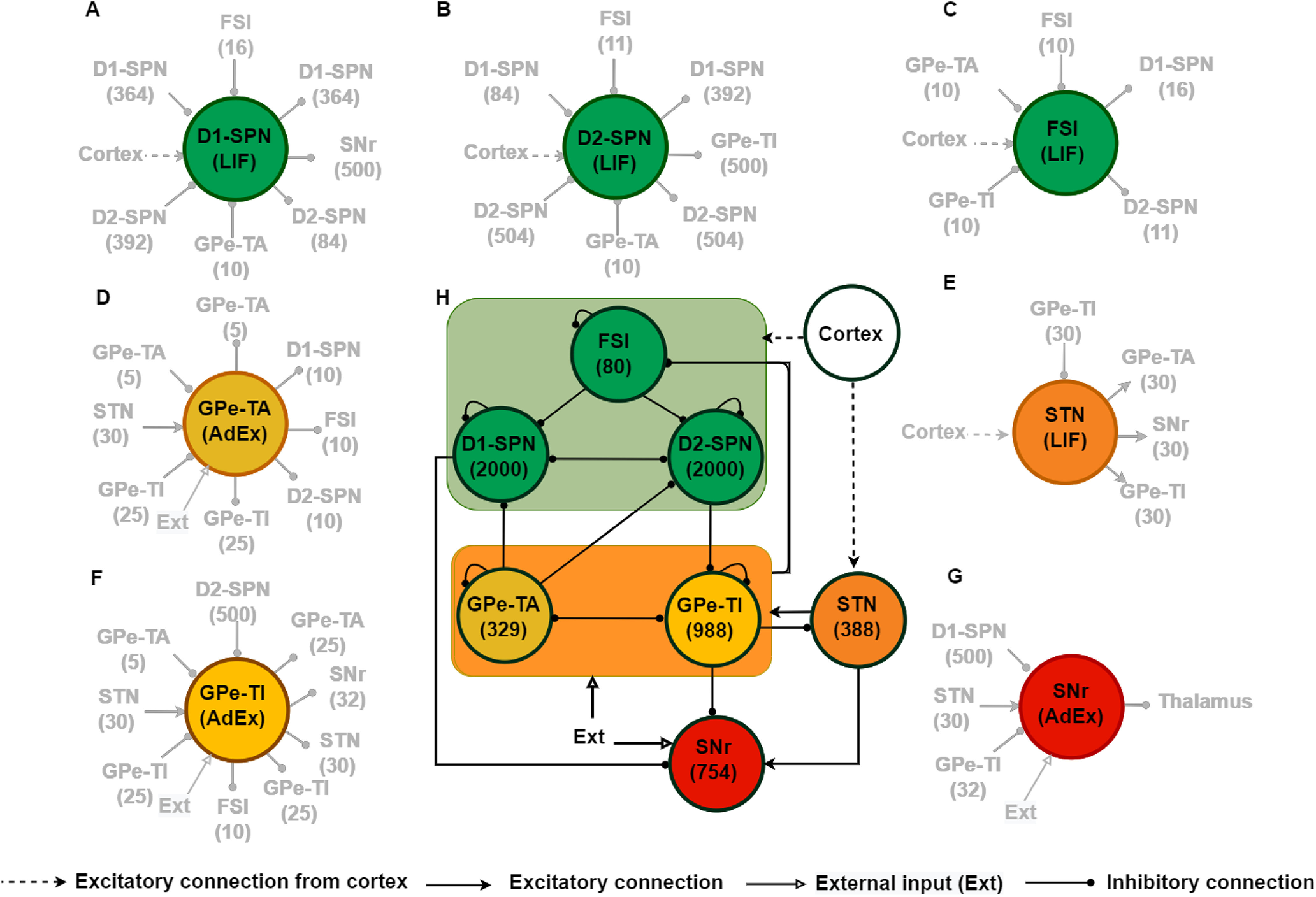
Schematic of the BG network model. ***A–G***, Schematic description of total number of inputs and outputs of a typical neuron in different subnetworks of the BG. ***H***, BG network structure along with the population size of individual nucleus. Within the BG network, the solid black lines with a circle at the end represent inhibitory synaptic connections and solid arrow lines represent excitatory synaptic connections. Dashed arrows denote the cortical excitatory input to BG.

In order to achieve a good trade-off between simulation efficacy and ability to capture the neuronal dynamics, we used two types of neuron models in our BG network. Striatal D1 and D2 type dopamine receptor expressing spiny neurons (D1-SPN and D2-SPN), fast-spiking interneurons (FSIs) and STN neurons were realized using the standard leaky-integrate-fire neuron (LIF) model with conductance-based synapses. The subthreshold dynamics of the membrane potential *V ^x^*(*t*) was governed by the Equation 1:

(1) $$C^{x}\frac{dV{\left(t\right)}^{x}}{dt}+G^{x}[V{\left(t\right)}^{x}-V_{rest}^{x}]=I^{syn}(t),$$

where *x* ∈ {D1-SPN, D2-SPN, FSI, and STN}. In Equation 1, *C^x^*, *G^x^*, *V_rest_* represent membrane capacitance, leak conductance and resting potentials, respectively. When *V^x^* reaches the threshold potential $V_{th}^{x}$, a spike is elicited and *V^x^* is reset to $V_{rest}^{x}$ for refractory duration *t_ref_* = 2 ms. *I^syn^*(*t*) models the total synaptic input current received by the neuron (see Fig. 1 for the various sources of inputs to these neurons).

All the parameter values for D1-SPN, D2-SPN, FSI and STN are summarized in the Tables 3, 4, 5, 8, respectively.

GPe-TA, GPe-TI and SNr neurons were modelled as a LIF neuron with exponential adaptation (AdEx) to capture rebound firing on release from hyperpolarization and spike trigerred adaptation as well (Nakanishi et al., 1987; Cooper and Stanford, 2000; Bugaysen et al., 2010). The subthreshold dynamics of these neurons were defined as:

(2) $$\begin{array}{l} C^{x}\frac{V{\left(t\right)}^{x}}{dt}=-G^{x}[V{\left(t\right)}^{x}-V_{rest}^{x}]+G^{x}\Delta_{T}exp(\frac{V{\left(t\right)}^{x}-V_{T}^{x}}{\Delta_{T}})-w^{x}+I^{syn}(t)\\ \tau_{w}{\dot{w}}^{x}=a(V{\left(t\right)}^{x}-V_{rest}^{x})-w^{x} \end{array},$$

where *x* ∈ {GPe-TA, GPe-TI, SNr}. In Equation 2, $V_{T}^{x}$ represents the spike-threshold, Δ_*T*_ represents the slope factor, *τ_w_* is the time constant of the adaptation variable *w*, and *a* controls the adaptation term. Given Equation 2, when *V ^x^* reaches the spike-cutoff potential then a spike is generated and *V ^x^*, as well as *w^x^* are reset at values *V_rest_*, *w^x^* + *b*, respectively, where *b* denotes the spike-triggered adaptation. *I^syn^*(*t*) models the total synaptic input current received by the neuron (see Fig. 1 for the various sources of inputs to these neurons).

The parameters for GPe-TA, GPe-TI and SNr neurons are given in the Tables 6, 7, 9, respectively.

Whether the response is shaped by the neuron complexity or network interactions has been highly debated without any clear conclusion (Prinz et al., 2004; Marder and Taylor, 2011; Sahasranamam et al., 2016). Here, we have chosen to use simplified models, so that we can exclusively focus on network interactions. Moreover, it should be noted that while LIF neuron model may appear simple, we can vary its inputs and parameters to fit many diverse input-output firing rate relationships (indeed, that is what we have done here).

### Synapse model

Neurons were connected using static conductance-based synapses. Each incoming spike elicited an α function shaped conductance transient, after a fixed delay since following the spike in the presynaptic neurons. The time course of the conductance transient was given as the following:

(3) $$g_{syn}^{x}(t)=\{\begin{array}{l} J_{syn}^{x}\frac{t}{\tau_{syn}}exp(\frac{-(t-\tau_{syn})}{\tau_{syn}}),\text{ for t}\geq 0\\ 0,\text{ for t}<0 \end{array},$$

where *syn* ∈ {exc, inh} and x∈D1−SPN,D2−SPN,FSI,GPe−TA,GPe−TI,STN,SNr. In Equation 3, $J_{syn}^{x}$ is the peak of the conductance transient and $\tau_{syn}^{x}$ is synaptic time constant. Each incoming synaptic current induces a current transient as given by the following:

(4) $$I_{syn}^{x}(t)=g_{syn}^{x}(t)[V^{x}(t)-V_{rev}^{x}],$$

where $V_{rev}^{x}$ is the reversal potential of the synapse for a neuron in population x∈{D1−SPN,D2−SPN,FSI,GPe−TA,GPe−TI,STN,SNr}. All synaptic parameters are specified in Table 2.

### BG network

The BG comprises of striatum, STN, GPe, SNr, and GPi in primates or entopeduncular nucleus (EPN) in rodents (Fig. 1). Although GPi and SNr are the output nuclei of the BG, but in this work, we only focus on the SNr activity. To model BG, we adapted a previously published model by Lindahl and Kotaleski (2016). However, unlike that model (Lindahl and Kotaleski, 2016), here, we reduced the time complexity of our proposed network by scaling down the size of striatum (D1-SPN, D2-SPN, FSI). Also a few synaptic and neural parameters were adjusted to achieve the network performance in healthy and PD conditions. The main differences between these two models are detailed in the later part of Materials and Methods.

Our reduced model of the BG consisted of 6539 neurons. Number of neurons in each subpopulation, number of connections and synaptic connectivity parameters are provided in Table 1.

**Table 1:** **Network and connection parameters (**Bahuguna et al., 2015**;** Lindahl and Kotaleski, 2016**)**

Name	Value	Description
*N_network_*	6539	Network size
$N_{network}^{D1-SPN}$	2000	Size of D1-SPN population
$N_{network}^{D2-SPN}$	2000	Size of D2-SPN population
$N_{network}^{FSI}$	80	Size of FSI population
$N_{network}^{STN}$	388	Size of STN population
$N_{network}^{GPe-TA}$	329	Size of GPe-TA population
$N_{network}^{GPe-TI}$	988	Size of GPe-TI population
$N_{network}^{SNr}$	754	Size of SNr population
$K_{D1-SPN}^{D1-SPN}$	364	Number of D1-SPN connections on each D1-SPN
$K_{D2-SPN}^{D1-SPN}$	84	Number of D1-SPN connections on each D2-SPN
$K_{D1-SPN}^{D2-SPN}$	392	Number of D2-SPN connections on each D1-SPN
$K_{D2-SPN}^{D2-SPN}$	504	Number of D2-SPN connections on each D2-SPN
$K_{D1-SPN}^{FSI}$	16	Number of FSI connections on each D1-SPN neuron
$K_{D2-SPN}^{FSI}$	11	Number of FSI connections on each D2-SPN neuron
$K_{D1-SPN}^{GPe-TA}$	10	Number of GPe-TA connections on each D1-SPN neuron
$K_{D2-SPN}^{GPe-TA}$	10	Number of GPe-TA connections on each D2-SPN neuron
$K_{FSI}^{FSI}$	10	Number of FSI connections on each FSI neuron
$K_{FSI}^{GPe-TA}$	10	Number of GPe-TA connections on each FSI neuron
$K_{FSI}^{GPe-TI}$	10	Number of GPe-TI connections on each FSI neuron
$K_{SNr}^{GPe-TI}$	32	Number of GPe connections on each SNr neuron
$K_{SNr}^{D1-SPN}$	500	Number of D1-SPN connections on each SNr neuron
$K_{SNr}^{STN}$	30	Number of STN connections on each SNr neuron
$K_{GPe-TI}^{D2-SPN}$	500	Number of D2-SPN connections on each GPe-TI neuron
$K_{GPe-TA}^{STN}$	30	Number of STN connections on each GPe-TA neuron
$K_{GPe-TI}^{STN}$	30	Number of STN connections on each GPe-TI neuron
$K_{GPe-TA}^{GPe-TA}$	5	Number of GPe-TA reciprocal connections
$K_{GPe-TI}^{GPe-TA}$	5	Number of GPe-TA connections on each GPe-TI neuron
$K_{GPe-TA}^{GPe-TI}$	25	Number of GPe-TI connections on each GPe-TA neuron
$K_{GPe-TI}^{GPe-TI}$	25	Number of GPe-TI reciprocal connections
$K_{STN}^{GPe-TI}$	30	Number of GPe-TI connections on each STN neuron

**Table 2:** Synaptic weight and delay parameters in healthy condition

Weight	Values (nS)	Delay	Values (ms)
$g_{D1-SPN}^{D1-SPN}$	−0.15 (Lindahl and Kotaleski, 2016)	$\Delta_{D1-SPN}^{D1-SPN}$	1.7
$g_{D2-SPN}^{D1-SPN}$	−0.375 (Lindahl and Kotaleski, 2016)	$\Delta_{D2-SPN}^{D1-SPN}$	1.7
$g_{D1-SPN}^{D2-SPN}$	−0.45 (Lindahl and Kotaleski, 2016)	$\Delta_{D1-SPN}^{D2-SPN}$	1.7
$g_{D2-SPN}^{D2-SPN}$	−0.35 (Lindahl and Kotaleski, 2016)	$\Delta_{D2-SPN}^{D2-SPN}$	1.7
$g_{D1-SPN}^{FSI}$	−2.6 (Bahuguna et al., 2015)	$\Delta_{D1-SPN}^{FSI}$	1.7
$g_{D2-SPN}^{FSI}$	−2.6 (Bahuguna et al., 2015)	$\Delta_{D2-SPN}^{FSI}$	1.7
$g_{D1-SPN}^{GPe-TA}$	−0.02	$\Delta_{D1-SPN}^{GPe-TA}$	7
$g_{D2-SPN}^{GPe-TA}$	−0.04	$\Delta_{D2-SPN}^{GPe-TA}$	7
$g_{FSI}^{FSI}$	−0.4	$\Delta_{FSI}^{FSI}$	1.7
$g_{FSI}^{GPe-TA}$	−0.25	$\Delta_{FSI}^{GPe-TA}$	7
$g_{FSI}^{GPe-TI}$	−1	$\Delta_{FSI}^{GPe-TI}$	7
$g_{SNr}^{GPe-TI}$	−52.5	$\Delta_{SNr}^{GPe-TI}$	3
$g_{SNr}^{D1-SPN}$	−15	$\Delta_{SNr}^{D1-SPN}$	7
$g_{SNr}^{STN}$	4.78	$\Delta_{SNr}^{STN}$	4
$g_{GPe-TI}^{D2-SPN}$	−1.08	$\Delta_{GPe-TI}^{D2-SPN}$	7
$g_{GPe-TA}^{STN}$	0.24	$\Delta_{GPe-TA}^{STN}$	2
$g_{GPe-TI}^{STN}$	0.175	$\Delta_{GPe-TI}^{STN}$	2
$g_{GPe-TA}^{GPe-TA}$	−0.11	$\Delta_{GPe-TA}^{GPe-TA}$	1
$g_{GPe-TI}^{GPe-TA}$	−1.3	$\Delta_{GPe-TI}^{GPe-TA}$	1
$g_{GPe-TA}^{GPe-TI}$	−0.35	$\Delta_{GPe-TA}^{GPe-TI}$	1
$g_{GPe-TI}^{GPe-TI}$	−1.3	$\Delta_{GPe-TI}^{GPe-TI}$	1
$g_{STN}^{GPe-TI}$	−0.3	$\Delta_{STN}^{GPe-TI}$	1

### Dopamine induced changes in neuron and synapse parameters

To model the effect of dopamine we followed the approach taken by Lindahl and Kotaleski (2016). Dopaminergic effects on SPNs, FSIs, STN, GPe and SNr neurons and their synaptic connections were modelled by modulating parameters such as the resting state potentials (*E_L_*), spike threshold (*V_th_*), and synaptic strengths. The dopamine modulation was modeled using a parameter *α_dop_* ranging between 0 (PD condition) and 1 (high dopamine). The normal state was mapped to *α_dop_* = 0.8 (*α_normal_*). The effect of the *α_dop_* on the neuron and synaptic properties are presented in the subsequent sections.

### Dopamine effects on neuron properties

In D1-SPNs, D1 type dopamine receptor activation not only shows a hyperpolarizing effect by increasing potassium inward rectifier (KIR) current, but also induces depolarizing effects on the resting membrane potential (Gruber et al., 2003). We modelled these two contributions by changing the spike threshold and resting membrane potential:

$$\begin{array}{l} V_{th}^{D1-SPN}=V_{th}^{D1-SPN}(1+\beta_{V_{th}}\phi )\\ E_{L}^{D1-SPN}=E_{L}^{D1-SPN}(1+\beta_{E_{L}}\phi ) \end{array},$$

where $\phi (\alpha_{dop})=\alpha_{dop}-\alpha_{normal}$. Thus, in dopamine depleted state both $V_{th}^{D1-SPN}$ and $E_{L}^{D1-SPN}$ were reduced. The parameters $\beta_{V_{th}}$ and $\beta_{E_{L}}$ (see Table 3) were chosen based on Humphries et al. (2009). Although Planert et al. (2013) suggested that dopamine concentration modulates the excitability of D2-SPN, in low-dopamine state no significant changes in their excitability were observed. Therefore, following the reasoning given by Lindahl and Kotaleski (2016) in this model we also ignored the effects of dopamine on the D2-SPNs. However, to test whether this assumption affects our results, we simulated dopamine induced change in D2-SPNs properties and measured the transient response (see Table 13). We confirmed that dopamine modulation of D2-SPN has a negligible effect on the transient response.

**Table 3:** D1-SPN neuron parameters (leaky integrate and fire model)

Name	Value	Description
V_reset	−87.2 mV (Gertler et al., 2008)	Reset value for v_m after spike
V_th	−45 mV (Bahuguna et al., 2015)	Spike threshold
tau_syn_ex	0.3 ms (Bahuguna et al., 2015)	Rise time of excitatory synaptic conductance
tau_syn_in	2 ms (Bahuguna et al., 2015)	Rise time of inhibitory synaptic conductance
E_L	−87.2 mV	Leak reversal potential
$\beta_{E_{L}}$	0.05	Magnitude of dopamine effect on resting potential
E_ex	0 mV	Excitatory reversal potential
E_in	−64 mV	Inhibitory reversal potential
I_e	128 pA	Constant input current
C_m	192 pF (Gertler et al., 2008)	Membrane capacitance
g_L	8.04 nS (Gertler et al., 2008)	Leak conductance
$\beta_{V_{th}}$	0.205	Magnitude of dopamine effect on threshold potential
t_ref	2 ms	Duration of refractory period

**Table 4:** D2-SPN neuron parameters (leaky integrate and fire model)

Name	Value	Description
V_reset	−85.4 mV (Gertler et al., 2008)	Reset value for v_m after spike
V_th	−45 mV (Bahuguna et al., 2015)	Spike threshold
tau_syn_ex	0.3 ms (Bahuguna et al., 2015)	Rise time of excitatory synaptic conductance
tau_syn_in	2 ms (Bahuguna et al., 2015)	Rise time of inhibitory synaptic conductance
E_L	−85.4 mV	Leak reversal potential
E_ex	0 mV	Excitatory reversal potential
E_in	−64 mV	Inhibitory reversal potential
I_e	0 pA	Constant input current
C_m	157 pF (Gertler et al., 2008)	Membrane capacitance
g_L	6.46 nS (Gertler et al., 2008)	Leak conductance
t_ref	2 ms	Duration of refractory period

We modelled the dopaminergic depolarizing effect induced through D1 type receptor activation on the FSIs, by modulating their resting membrane potential:

$$E_{L}^{FSI}=E_{L}^{FSI}(1+\beta_{E_{L}}\phi ),$$

where $\beta_{E_{L}}$ (see Table 5) was set such that $E_{L}^{FSI}$ at low dopamine level was 5 mV lower than that of the high dopamine level (Bracci et al., 2002).

**Table 5:** FSI neuron parameters (leaky integrate and fire model)

Name	Value	Description
V_reset	−65 mV (Klaus et al., 2011)	Reset value for v_m after spike
V_th	−54 mV (Bahuguna et al., 2015)	Spike threshold
tau_syn_ex	0.3 ms (Bahuguna et al., 2015)	Rise time of excitatory synaptic conductance
tau_syn_in	2 ms (Bahuguna et al., 2015)	Rise time of inhibitory synaptic conductance
E_L	−65 mV	Leak reversal potential
E_ex	0 mV	Excitatory reversal potential
E_in	−76 mV	Inhibitory reversal potential
I_e	0 pA	Constant input current
C_m	700 pF (Klaus et al., 2011)	Membrane capacitance
g_L	16.67 nS (Russo et al., 2013)	Leak conductance
$\beta_{E_{L}}$	−0.078 (Lindahl and Kotaleski, ;2016)	Magnitude of dopamine effect on resting potential
t_ref	2 ms	Duration of refractory period

The dopaminergic depolarizing effects on the GPe neurons (both TA and TI) are manifested as up-regulation of the hyperpolarization-activated cyclic nucleotide-gated (HCN) channels (Chan et al., 2011) which essentially results in a change in the resting membrane potential of the neurons. To mimic this effect, we changed the resting membrane potential of the GPe neurons in the following manner:

$$E_{L}^{GPe}=E_{L}^{GPe}(1+\beta_{E_{L}}\phi ).$$

The values of $\beta_{E_{L}}$ for both the GPe-TA and GPe-TI neurons (see Tables 6, 7) were set such that the resting state potential of the GPe neurons at low dopamine level was 10 mV lower than that of its value at high dopamine level.

**Table 6:** GPe**-TA neuron parameters (**Lindahl and Kotaleski, 2016**; adaptive exponential integrate and fire model)**

Name	Value	Description
a	2.5 nS	Subthresholded adaption
b	105 pA	Spike triggered adaption
$\beta_{E_{L}}$	−0.181	Magnitude of dopamine effect on resting potential
Δ*_T_*	2.55 ms	Slope factor
tau_w	20 ms	Adaption time constant
V_reset	−60 mV	Reset value for v_m after spike
V_th	−54.7 mV	Spike initiation threshold
tau_syn_ex	1 ms	Rise time of excitatory synaptic conductance
tau_syn_in	5.5 ms	Rise time of inhibitory synaptic conductance
E_L	−55.1 mV	Leak reversal potential
E_ex	0 mV	Excitatory reversal potential
E_in	−65 mV	Inhibitory reversal potential
I_e	1 pA	Constant input current
C_m	60 pF	Membrane capacitance
g_L	1 nS	Leak conductance
t_ref	2 ms	Duration of refractory period

**Table 7:** **GPe-TI neuron parameters (** Lindahl and Kotaleski, 2016 **; adaptive exponential integrate and fire model)**

Name	Value	Description
a	2.5 nS	Subthresholded adaption
b	70 pA	Spike triggered adaption
$\beta_{E_{L}}$	−0.181	Magnitude of dopamine effect on resting potential
Δ*_T_*	1.7 ms	Slope factor
tau_w	20 ms	Adaption time constant
V_reset	−60 mV	Reset value for v_m after spike
V_th	−54.7 mV	Spike initiation threshold
tau_syn_ex	4.8 ms	Rise time of excitatory synaptic conductance
tau_syn_in	1 ms	Rise time of inhibitory synaptic conductance
E_L	−55.1 mV	Leak reversal potential
E_ex	0 mV	Excitatory reversal potential
E_in	−65 mV	Inhibitory reversal potential
I_e	12 pA	Constant external input current
C_m	40 pF	Membrane capacitance
g_L	1 nS	Leak conductance
t_ref	2 ms	Duration of refractory period

**Table 8:** STN neuron parameters (leaky integrate and fire model)

Name	Value	Description
V_reset	−70 mV (Lindahl and Kotaleski, 2016)	Reset value for v_m after spike
V_th	−64 mV (Lindahl and Kotaleski, 2016)	Spike threshold
tau_syn_ex	0.33 ms	Rise time of excitatory synaptic conductance
tau_syn_in	1.5 ms	Rise time of inhibitory synaptic conductance
E_L	−80.2 mV (Lindahl and Kotaleski, 2016)	Leak reversal potential
E_ex	−10 mV	Excitatory reversal potential
E_in	−84 mV	Inhibitory reversal potential
I_e	1 pA	Constant input current
C_m	60 pF (Lindahl and Kotaleski, 2016)	Membrane capacitance
g_L	10 nS (Lindahl and Kotaleski, 2016)	Leak conductance
t_ref	2 ms	Duration of refractory period

Dopaminergic effect on the SNr neurons (Zhou et al., 2009) was realized by changing their resting membrane potential:

$$E_{L}^{SNr}=E_{L}^{SNr}(1+\beta_{E_{L}}\phi ),$$

where $\beta_{E_{L}}$ (see Table 9) was taken such that the resting potential at low dopamine level was 5 mV lower than its value at high dopamine level.

**Table 9:** SNr neuron parameters (adaptive exponential integrate and fire model)

Name	Value	Description
a	3 nS (Lindahl and Kotaleski, 2016)	Subthresholded adaption
b	200 pA (Lindahl and Kotaleski, 2016)	Spike triggered adaption
$\beta_{E_{L}}$	–0.0896 (Lindahl and Kotaleski, 2016)	Magnitude of dopamine effect on resting potential
Δ*_T_*	1.6 ms	Slope factor
tau_w	20 ms (Lindahl and Kotaleski, 2016)	Adaption time constant
V_reset	−65 mV (Lindahl and Kotaleski, 2016)	Reset value for v_m after spike
V_th	−55.2 mV (Lindahl and Kotaleski, 2016)	Spike initiation threshold
tau_syn_ex	5.7 ms	Rise time of excitatory synaptic conductance
tau_syn_in	2.04 ms	Rise time of inhibitory synaptic conductance
E_L	−55.8 mV (Lindahl and Kotaleski, 2016)	Leak reversal potential
E_ex	0 mV	Excitatory reversal potential
E_in	−80 mV	Inhibitory reversal potential
I_e	0 mV	Constant external input current
C_m	80 pF (Lindahl and Kotaleski, 2016)	Membrane capacitance
g_L	3 nS (Lindahl and Kotaleski, 2016)	Leak conductance
t_ref	2 ms	Duration of refractory period

The scaling factors $\beta_{i}(i\in \{E_{L},V_{th}\}$), for the linear modulation ( $\phi (\alpha_{dop})=\alpha_{dop}-\alpha_{normal}$) were tuned for each parameter to match their experimentally reported results (Lindahl and Kotaleski, 2016) in both normal and PD conditions (rodent models).

### Dopamine effects on synaptic weights

High dopamine strengthens cortical projection on to D1-SPN and weakens cortical projections on to D2-SPN (Hernández-Echeagaray et al., 2004). The decrease in connectivity both in terms of synaptic strength and number of recurrent connections among SPNs is also attributed to dopamine depletion (Taverna et al., 2008). In addition, dopamine depletion is reported to enhance the strength of GABAergic synapses (Bracci et al., 2002) between FSI-FSI and increases the number of connections between FSI and D2-SPN (Gittis et al., 2011), but not D1-SPN. Within the GPe, dopamine depletion strengthens the GPe ↔GPe (Miguelez et al., 2012) and GPe →FSI connections (Bracci et al., 2002). In addition to that, it also strengthens the GPe-TA →SPN synapses (Glajch et al., 2016).

Dopamine depletion also strengthens the D2-SPN projections on to GPe neurons through reduced D2-receptor activation (Chuhma et al., 2011). Similarly, reduced dopamine concentration strengthens the STN →GPe synapses (Hernández et al., 2006) and also responsible for increasing the synaptic efficacy in GPe-TI →STN synapses (Baufreton and Bevan, 2008). Galvan and Wichmann (2008) and Chu et al. (2017) claimed that cortico-STN transmission is reduced because of dopamine loss, but, Shen and Johnson (2000) suggested strengthening of the cortico-STN synapses and enhancement of the responsiveness of cortico-STN-SNr pathway at low dopamine level. Experimental data (Kita and Kita, 2011; Sano and Nambu, 2019; Chiken et al., 2021; Wahyu et al., 2021) also reported that the strength of early excitation zone of the transient response in PD condition is either comparable to or much stronger than healthy state. This could be caused either by strengthening of cortico-STN synapses keeping STN →SNr synaptic property unchanged or by weakening of the cortico-STN synapses but increasing the STN to SNr weight. As dopamine receptor D1 and D2 activation induce two opposing effects, i.e., facilitating and depressing, respectively, on STN–SNr EPSC (Ibáñez-Sandoval et al., 2006), it is not well understood how dopamine depletion affects the same in PD state (Lindahl and Kotaleski, 2016). Thus, in our model, we did not change the strength of STN →SNr synapse in low dopamine state and modeled the dopamine depletion induced changes in cortico-STN synapse by increasing the synaptic weight (Holgado et al., 2010; Lindahl and Kotaleski, 2016). On the other hand, at low dopamine, the D1-SPN to SNr connection strength was reduced (Chuhma et al., 2011), therefore, *I_GABA_* from D1-SPN to SNr was modelled to reflect the same.

Dopaminergic effect on the synaptic strength ( $g_{syn}^{x\to y}$) was modelled as $g_{syn}^{x\to y}=g_{syn}^{x\to y}(1+\beta_{y}^{x}\phi )$, where *x*, *y* ∈ {FSI, D1-SPN, D2-SPN, STN, Cortex, GPe, SNr} and the values of $\beta_{y}^{x}$ were given in the Table 10.

**Table 10:** Synaptic dopamine parameters

Name	Value in PD-biphasic	Value in PD-triphasic
$\beta_{FSI}^{FSI}$	−1.27 (Lindahl and Kotaleski, 2016)	−1.27
$\beta_{FSI}^{GPe}$	−0.53 (Lindahl and Kotaleski, 2016)	−0.53
$\beta_{GPe}^{GPe}$	−0.83 (Lindahl and Kotaleski, 2016)	−0.83
$\beta_{GPe-TI}^{D2-SPN}$	−1.00	**−0.48**
$\beta_{GPe}^{STN}$	−0.3	−0.3
$\beta_{D1-SPN}^{Cortex}$	1.04 (Lindahl and Kotaleski, 2016)	1.04
$\beta_{D2-SPN}^{Cortex}$	−0.26 (Lindahl and Kotaleski, 2016)	−0.26
$\beta_{D2-SPN}^{FSI}$	−0.90 (Lindahl and Kotaleski, 2016)	−0.90
$\beta_{SPN}^{SPN}$	0.88 (Lindahl and Kotaleski, 2016)	0.88
$\beta_{D1-SPN}^{GPe-TA}$	−1.22 (Lindahl and Kotaleski, 2016)	−1.22
$\beta_{D2-SPN}^{GPe-TA}$	−1.15 (Lindahl and Kotaleski, 2016)	−1.15
$\beta_{SNr}^{D1-SPN}$	0.42	**0.56** (Lindahl and Kotaleski, 2016)
$\beta_{STN}^{Cortex}$	−1.15	−1.15
$\beta_{STN}^{GPe}$	−0.54	**−0.24** (Lindahl and Kotaleski, 2016)

To obtain a triphasic response in PD condition, we had to change a few parameters of the network tuned in default PD state (biphasic). These changes are marked in boldface.

### External inputs

In our network model, all the neuronal populations received uncorrelated excitatory Poisson input spike-train. This input was provided to obtain baseline firing rate in the neurons. For the striatum this input corresponds to the cortical and thalamic inputs. For the STN, this input corresponds to the cortical inputs. For the GPe and SNr neurons, this input may correspond to either the endogenous activity or other inputs from outside the BG. Each neuron in a given population received a different realization of Poisson type spikes with the same parameters. The input rates were tuned both in normal and PD conditions to ensure that the basal firing rates (FR) of different subnuclei were consistent with the *in vivo* recordings in anaesthetized rats. For example, in normal condition baseline firing rate (in Hz) of D1-SPN and D2-SPN ∈ [0.01, 2.0] (Miller et al., 2008; Lindahl and Kotaleski, 2016), FSI ∈ [10, 20] (Gage et al., 2010), STN ∈ [10, 13] (Fujimoto and Kita, 1993; Paz et al., 2005), and SNr [20, 35] (Kita and Kita, 2011; Benhamou and Cohen, 2014). The baseline activities of GPe-TA (11.8 ± 1.1 Hz) and GPe-TI (24.2 ± 0.7 Hz) is consistent with experimental data Mallet et al. (2008, 2012). Similarly, in PD condition, frequencies of the background noise (in Hz) were also tuned to achieve range of basal firing rate of D1-SPN ∈ [0.1, 0.5], D2-SPN ∈ [1, 2], GPe-TA ∈ [12, 16] (de la Crompe et al., 2020), GPe-TI ∈ [17, 20] (de la Crompe et al., 2020), and STN ∈ [26, 29] (de la Crompe et al., 2020). For SNr, Sano and Nambu (2019) claimed a decrease of basal firing rate in PD conditions; however, others (Ruskin et al., 2002; Kita and Kita, 2011; Wahyu et al., 2021) had not observed firing rate changes in PD state. Given this, we kept basal firing rate of SNr the same as it is in normal state.

To characterize the effect of a transient cortical stimulation on the neuronal responses of the SNr, we stimulated striatal and STN neurons with a brief stimulus which amounted to injection of a rate modulated Poisson spike-train (Fig. 3, top panel). The fraction of stimulated neurons is specified in corresponding figures in Results.

This input was modelled by using the inhomogeneous_poisson_generator device in NEST (Gewaltig and Diesmann, 2007). Because the transient stimulation was modelled as injection of Poisson spike-train for a brief period of time, we could control the strength of input stimulation by varying the amplitude of the EPSP generated by the injected spike train. Moreover, this allowed us to modulate the strength of input in relation to dopamine levels (see above, Dopamine effects on synaptic weights, for how dopamine affected synaptic weights). Transient response was measured in both normal and PD conditions.

### Main differences between our model and the one by Lindahl and Kotaleski (2016)

Here we build on the model by Lindahl and Kotaleski (2016); however, we made a few changes in the neuron and synapse models and changed the number of neurons in some of the BG subnetworks. The main focus of our work is to investigate how the structure of connectivity within and between BG subnetworks. Therefore, it was important to reduce the model complexity when possible. Unlike their model (Lindahl and Kotaleski, 2016), striatal and STN neurons were modelled as simple LIF neurons without any kind of adaptation and, all the synapses were static as opposed to the dynamic ones. As we have argued later, this simplification has no specific effect on our key results. Striatal SPNs spike at low firing rate in an asynchronous manner, despite their recurrent connectivity and inputs. It is not necessary to model the striatum with ≈75,000 neurons as was done by Lindahl et al. (2013). When the parameters are appropriately scaled, we can obtain low firing rate asynchronous activity in a network of 4000 neurons. Therefore, we also reduced the size of striatal neuronal population. To this end, we changed the synaptic strengths and a few neuronal model parameters, such that the average synaptic input to GPe and GPi/SNr neurons was identical to the model used by Lindahl and Kotaleski (2016). This ensured that the model had the same repertoire of dynamical states as that of the model by Lindahl and Kotaleski (2016). Finally, to generate triphasic shaped transient responses, we also changed the values of $\beta_{y}^{x}(\phi )$ (see Table 10). Besides these changes, we followed the model closely while modeling the effects of dopamine on neuron and synapse parameters.

### Limitations of the model

Unlike in the experimental data, in our model, all neurons responded with similar response profile. This is because the model is homogeneous in terms of neuron and synapse properties. It was important to keep the model homogeneous to isolate the various interactions that lead to triphasic or other shapes of transient response. Furthermore, all synapses are static in this model. We note that Lindahl et al. (2013) suggested that synaptic short term plasticity is important for the triphasic response when STN is stimulated. However, as we show in this study, triphasic responses do not require synaptic short-term dynamics. Moreover, to the best of our knowledge, there is no experimental evidence for short-term plasticity to be the cause of triphasic response. Furthermore, given that the short-term dynamics time constants are of the order of 100 ms, the effects of short-term dynamics may not be strong in the β band. It is possible that short-term dynamics of synapses may affect the properties of β band bursts but at least in this study we do not investigate such transient oscillation.

We have only considered effects of changes in the dopamine baseline. Transient responses of BG could also be accompanied by phasic change in DA levels. Such effects have been ignored. We did not explicitly model the effect of low-dopamine on D2-SPNs by changing the neuron and synapse properties. Instead, we mimicked this effect indirectly by increasing their baseline activity in PD condition. Next, we have only modelled the FSIs and ignored other types of interneurons. Only recently a detailed microcircuit has been modelled with numerical simulations (Hjorth et al., 2020). In future studies it may be possible to use a reduced version of that network for BG modeling. Finally, our model does not address the changes in the spatiotemporal dynamics of BG nuclei given cortical stimulation, as connectivity within each subnetwork is independent of spatial distances among the neurons.

### Simulation tools

All the simulations were performed using the simulator NEST 2.20 (Jordan et al., 2019). All differential equations were integrated using Runga–Kutta method with a time step of 0.1 ms.

### Code accessibility

The code to simulate the BG network is available on GitHub: https://github.com/arvkumar/Basal-Ganglia-Transient-Response. The *Readme.ṫxt* file provides the necessary instructions to run the code. The simulation code was written in Python 3.0 and requires NEST 2.20 (Jordan et al., 2019) to run. The code is also provided as Extended Data 1 file.

10.1523/ENEURO.0376-21.2022.ed1 Extended Data 1 Basal ganglia transient response. Download Extended Data 1, ZIP file.

### Data analysis

#### Transient response analysis

To get better estimate of the transient response we performed 100 trials and recorded the response over 1200 ms. The onset of the transient input (*T_stimulation_*) was randomly chosen between 700 and 900 ms for every trial. Note that, the stimulation point was chosen between 700 and 900 ms to discard the initial transient that appears at the beginning of the simulation. To understand the effect of the transient stimulation on the SNr activities, the neuronal responses of SNr neurons were observed before and after the cortical stimulation point *T_stimulation_*. A 350-ms window size was defined around *T_stimulation_* to extract responses from each trial. For this, we used a time window of 100 ms before and 250 ms after the stimulation point. *T_stimulation_* is marked as 0 in the figures mentioned in the subsequent sections.

The responses were evaluated by constructing peristimulus time histograms (PSTH), using 1-ms rectangular bins for each trial data. The baseline neuronal activity was computed based on the mean and SD of the PSTH data for 100-ms duration before the onset of the stimulation point.

**Figure 2.**
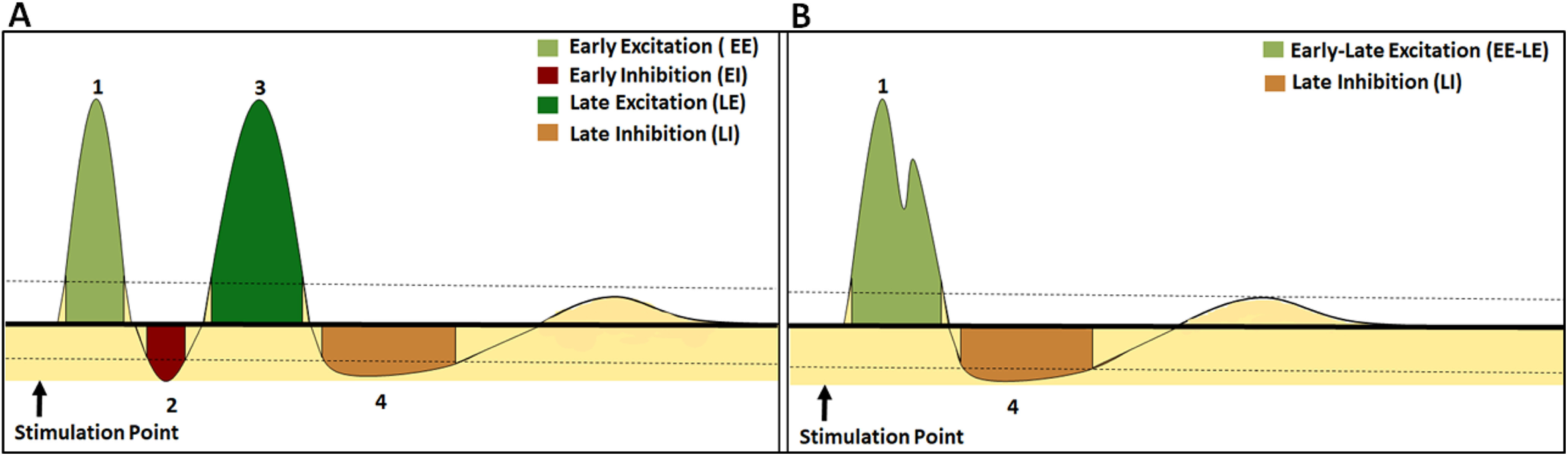
Characterization of transient responses in the SNr. ***A***, A schematic representation of cortical stimulation induced so called triphasic response patterns in the SNr (seen in the healthy state). The triphasic response consists of early excitation (EE), early inhibition (EI), late excitation (LE), and a late inhibition (LI). ***B***, A schematic representation of biphasic shaped transient response patterns in the SNr (corresponding to what is seen in PD condition). It consists of EE, LE, and a LI. The horizontal bold line and two dotted lines denote the prestimulus mean (basal) firing rate and 95% confidence interval, respectively.

Visual inspection of the experimental measurement of the transient responses shows it has actually four distinct phases (schematically shown in Fig. 2). However, strangely in the literature such response has been referred to as triphasic. Here, to analyze the transient response of the SNr, both in normal and PD conditions, the PSTH were divided into four zones (see Fig. 2) based on the change in firing rate. These zones consisted of two excitatory (EE and LE for early and late, respectively) and two inhibitory (EI and LI) zones. The change, i.e., increase or decrease in firing activity was marked as excitation or inhibition, respectively, if the firing rate was significantly above or below the baseline (*p *<* *0.05, one-tailed Z-test) discharge rate for at least two consecutive time bins (2 ms; Sano et al., 2013). The latency of each zone was measured as the time when the first bin exceeded the baseline. Similarly, the zone terminated when activities during two consecutive bins fell below the significance level. The end time was determined as the time of the last bin exceeding the significance level. The total time duration from the first bin to the last bin (of a significant response) was considered as the duration of each zone. The sum of heights of bins within a particular zone is considered as the area as well as the strength of the zone, whereas the area per unit time (area/time) indicates the average strength of that zone.

Thus, we extracted the following features from PSTH for each zone: latency (*L*), duration (*D*), absolute area indicating strength (*A*) of that zone, mean (*H_μ_*), and SD (*H_σ_*) of bin heights. In addition, we also measured the peak amplitudes (*H_p_*) of each zone (i.e., $H_{p}\in \{H_{max}^{EE},H_{max}^{LE},H_{min}^{EI},H_{min}^{LI}\}$).

Finally, *F_i_* (i ∈{EE, EI, LE, LI}) is a six-dimensional vector ({*L*, *D*, *A*, $H_{\mu },\;H_{\sigma }$, *H_p_*}). *F_i_*s were computed for each zone in different network conditions (F for all four zones). Such networks, for example, were simulated by restoring selected synaptic strengths from PD condition to the normal condition. These can be referred to as *TestNetworks*. The similarity between such a *TestNetwork* and networks tuned in healthy condition and PD condition was calculated using Euclidean distance metric:

(5) $$\begin{array}{l} Dist_{TestNetwork}^{REF}=\sqrt{\sum_{k=1}^{24}{\left(F^{k}(REF)-F^{k}(TestNetwork)\right)}^{2}}\\ \left(whereREF=\{normal,PD\}\right) \end{array}.$$

In order to observe the statistical variation of the above features within the normal and PD conditions, a subpopulation of SNr neurons was considered. From the whole population of SNr neurons, a percentage of neurons (*N_S_*%) was randomly chosen to represent an observation. The responses of these subpopulation of SNr neurons were averaged over multiple trials (*O_S_* = 100 in the present simulation) and then the features of the four zones were extracted. The choice of the subpopulation of *N_S_*% was also varied for every observation and a large number of such observations (*O_S_*) were made. These observations were used to derive the mean and SD of the features for each of the above-mentioned EE, EI, LE, and LI zones. Here, we have considered *O_S_* = 100 and *N_S_* = 50%.

In experimental studies (Ozaki et al., 2017; Sano and Nambu, 2019), in healthy state, the transient response is often characterized by dividing the response in three zones EE, EI, and LE. In low-dopamine state the experimentally observed biphasic response pattern could consist of any two zones out of the three zones (EE, EI, and LE; Sano and Nambu, 2019; Wahyu et al., 2021). Experimental data also shows that in both normal and PD conditions, LE is followed by an LI zone (Kita and Kita, 2011). Therefore, here we defined four zones to characterize the transient response in the healthy state (Fig. 2*A*). Thus, although we have defined four zones, we still refer to it as a triphasic response to be consistent with the terminology used in the literature. In our simulations, biphasic response (Fig. 2*B*) observed in PD condition consisted of EE and LE, while the EI zone was not observed. We merged the EE and LE zones together for computing the features of the PD-biphasic response.

### Global network activity

The oscillatory behavior of population activities was assessed in PD as well as normal conditions. We ran the simulation for longer duration (5 s), without any transient input, to allow the oscillations to set in to their steady state. This experiment was also conducted over 100 trials.

Synchrony in the firing rates of a neuronal population was estimated using Fano factor (*FF_pop_*; Kumar et al., 2011):

(6) $$FF_{pop}=\frac{V_{pop}}{E_{pop}},$$

where *E_pop_* and *V_pop_* are the mean and variance of the neuronal activity for the same population, respectively. For an uncorrelated ensemble of Poisson processes, *FF_pop_* = 1 and when neurons tend to correlate, *FF_pop_* > 1. Here, we binned the neuronal activity using rectangular bins of 3-ms duration. This window size was similar to the one used in previous studies (Mallet et al., 2008; Lindahl and Kotaleski, 2016).

To determine the strength of oscillatory neuronal activities in the β band, we estimated the oscillation index (*OI_pop_*). To this end, we estimated the spectrum of the population activity [*S_pop_*(*f*)]. As we used 3 ms bins to calculate the PSTH, the sampling frequency (*F_s_*) was 333.3 Hz. To estimate the oscillation index, we measured the relative power confined in the β band:

(7) $$OI_{pop}=\frac{\int_{12}^{30}S_{pop}(f)df}{\int_{0}^{F_{s}/2}S_{pop}(f)df}.$$

The phase relationships of the firing patterns between the two types (GPe-TA and GPe-TI) of GPe nuclei as well as with STN were computed from the PSTH, having bin size of 1 ms. As we were interested in analyzing the pathologic β oscillation, the individual PSTH responses were bandpass filtered between 12 and 30 Hz.

Initially, at every time instance, corresponding to each bin, the instantaneous phase was calculated using the Hilbert transform. Then the differences of the instantaneous phases were obtained between a pair of nuclei, for every 1 ms. Finally, the histogram of the difference in the phase was obtained with 100 bins in the range of 0 to *π*.

## Results

The standard feedforward model of the BG (Albin et al., 1989) predicts that transient cortical stimulation will result in what has been denoted as the triphasic response in the SNr as the stimulus induced activity is propagated over the hyperdirect, direct and indirect pathways. Indeed, many neurons, at least in a healthy state, do show a triphasic response *in vivo*. However, in both healthy and dopamine-depleted conditions, response pattern of a sizeable fraction of neurons deviates from the triphasic response shape (Kita and Kita, 2011; Sano and Nambu, 2019; Chiken et al., 2021; Wahyu et al., 2021) indicating the role of recurrent interactions within and between BG nuclei. To understand how different neurons and network parameters shape the output of SNr when the striatum and STN are transiently stimulated, we used numerical simulations of the BG network with spiking neurons. In the model, we systematically varied the dopamine level and studied how strength of different connections in the BG affects the shape of the transient response in both healthy and PD conditions. Here, we set the dopamine level to 0.8 and 0.0 to tune the model into healthy and PD conditions, respectively (Lindahl and Kotaleski, 2016).

### Cortically evoked transient response in SNr

**Figure 3.**
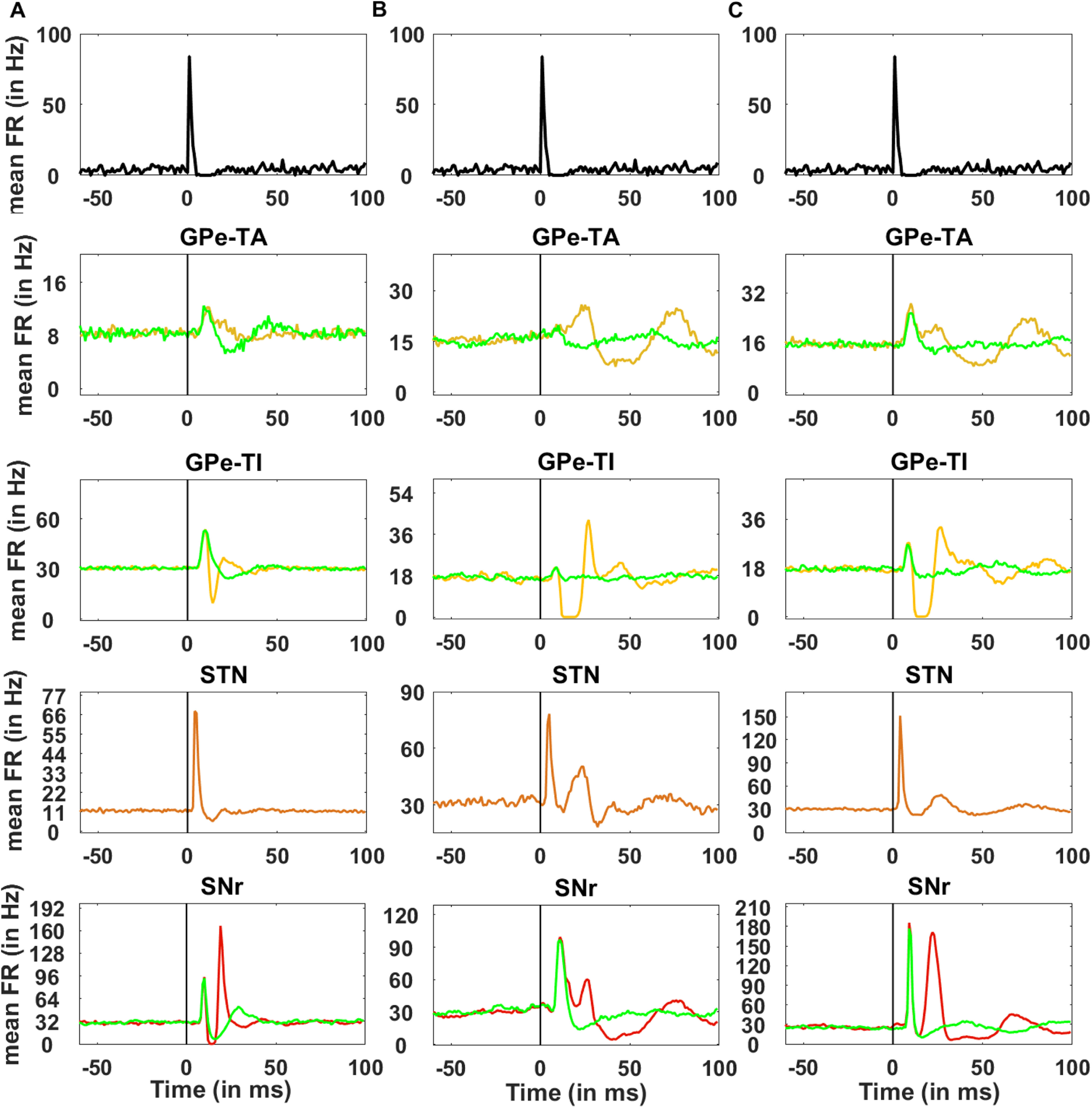
Cortically evoked responses in the GPe-TA, GPe-TI, STN, and SNr. Cortical input (top row) was given to 50% striatal and STN neurons. The rate of cortical input as a function of time (top row) was identical across trials however, each stimulated neuron received a different realization of spikes. FR denotes the firing rate. ***A***, Average PSTH (100 trials) of all neurons in GPe-TA, GPe-TI, STN, and SNr in normal condition. ***B***, Average PSTH (100 trials) of all neurons in GPe-TA, GPe-TI, STN, and SNr in PD-biphasic condition. ***C***, Average PSTH (100 trials) of all neurons in GPe-TA, GPe-TI, STN, and SNr in PD-triphasic condition. The black vertical line represents the stimulation onset. Each population is assigned a different color and the green curve in each panel denotes the transient response of neurons in response to only STN stimulation. Here, 0 ms denotes the stimulation onset. For spike activity rasters, please see the Extended Data Figure 3-1.

Transient stimulation of the neocortex results in a brief excitation followed by inhibition caused by the recurrent inhibition. Therefore, we stimulated the striatal and STN neurons with a rate modulated Poisson process that mimicked the excitation-inhibition pattern cortical stimulus response (see Fig. 3; Materials and Methods). Consistent with the predictions of a feedforward model of the BG and *in vivo* experimental data, in healthy state SNr neurons responded with a triphasic response consisting of early excitation (because of STN), inhibition (because of the D1-SPN projections), and late excitation (because of indirect pathway), i.e., the EE-EI-LE response (see Fig. 3*A*). By contrast, in PD condition, SNr neurons responded with a biphasic response (from here on referred to as default PD condition), consisting of a prominent early excitation and late excitation (i.e., EE-LE; see Fig. 3*B*). Thus, the model suggests that persistent dopamine depletion (see Materials and Methods; Table 10) not only affects the steady-state of the BG network (i.e., β band oscillations) but also impair the transient inhibitory effect of the striatal inputs to the SNr because of weak D1-SPN projections as well as stronger activity along the indirect pathway. In PD conditions, the missing EI phase is attributed to the weaker D1-SPN →SNr connection and the stronger inhibitory influence of the D2-SPN →GPe-TI connection which disinhibited the SNr neurons and subsequently resulted in the prolonged LE phase. In fact, in our simulations, in extreme PD conditions (when *α_dop_* = 0) D1-SPN response was almost zero.

10.1523/ENEURO.0376-21.2022.f3-1 Extended Data Figure 3-1 Raster plot of cortically evoked responses in the GPe-TA, GPe-TI, STN, and SNr. Raster plot is generated using only single trial data. ***A***, Raster plot of all neurons in GPe-TA, GPe-TI, STN, and SNr in normal condition. ***B***, Same as ***A*** but in PD-biphasic condition. ***C***, Same as ***A*** but in PD-triphasic condition. The black vertical line represents the stimulation onset. Here, 0 ms denotes the stimulation onset. Download Figure 3-1, TIF file.

Experimental data show that even in the PD condition, 15–40% of the SNr neurons respond in a triphasic manner (Sano and Nambu, 2019; Wahyu et al., 2021). In our model, to generate a triphasic response in PD condition (Fig. 3*C*), we needed to make additional changes other than those brought in by low dopamine. In particular, we decreased D2-SPN →GPe-TI, increased D1-SPN →SNr, and decreased GPe-TI →STN connections (see Table 10 for numerical values). Note that, although these synaptic changes in the network parameters affected the shape of the transient response but did not affect the oscillation and synchrony in both the PD states (OI for PD-biphasic = 0.24 and PD-triphasic = 0.23, FF for PD-biphasic = 18.01 and PD-triphasic = 13.49; see Fig. 8 for more details). In other words, small changes in the synaptic connectivity can affect the transient response without changing the baseline activity in a qualitative manner.

In the above, we ignored that dopamine may also affect the properties of D2-SPNs and thereby may affect the transient response. To check whether dopamine dependent modulation of D2-SPNs may affect the transient response we changed the excitability of D2-SPNs as a function of dopamine levels (Day et al., 2008; Damodaran et al., 2015). However, these changed in D2-SPNs did not affect the shape of the transient response (see Table 13) in either of the two PD states.

**Figure 4.**
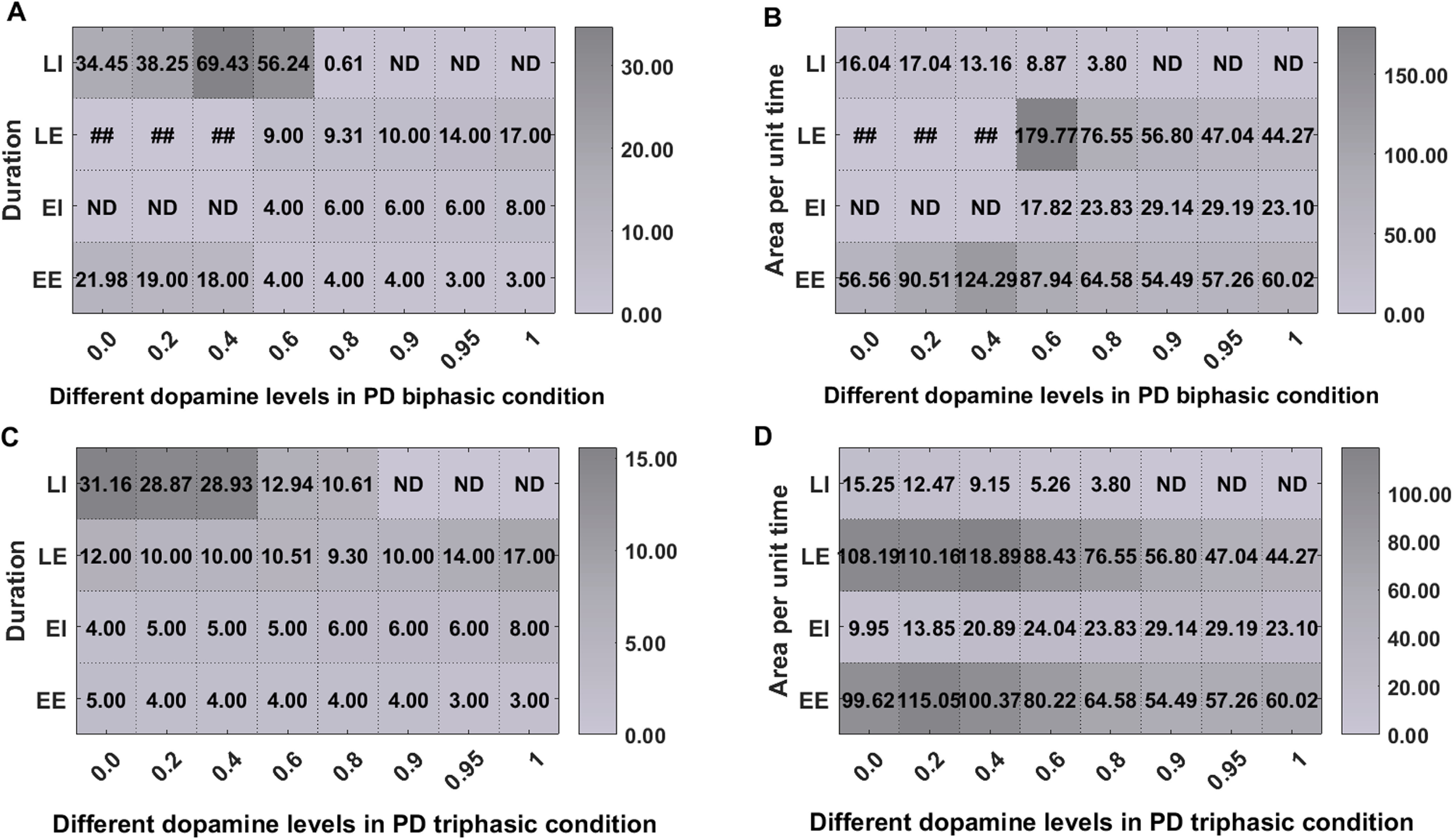
Effect of different dopamine levels (0.0–1) on the shape of transient response in the SNr. ***A***, Changes in the duration of the four zones of the transient response for different dopamine levels in PD-biphasic condition. ***B***, Changes in the area per unit time (area/time) of the four zones of the transient response for different dopamine levels in PD-biphasic condition. ***C***, Same as ***A*** but for in PD-triphasic condition. ***D***, Same as ***B*** but for in PD-triphasic condition. ##: The duration and area/time corresponding to dopamine level 0.0, 0.2, and 0.4 in PD-biphasic state are given for the complete excitatory response comprising of both EE and LE zones. In these cases, the EI was not detectable using statistical test, hence the two excitations (EE and LE) were merged during computation of the parameters. ND denotes that the zone was not detected using significance test (see Materials and Methods, Data analysis).

Next, we also tested how progressive change in the dopamine levels may affect the shape of the transient response. To this end, we tuned the model in either PD biphasic or PD triphasic state and systematically increased the level of dopamine. We found that the four phases of the transient response change gradually as a function of dopamine level (Fig. 4*A*,*B*) in PD-triphasic condition. By contrast, in PD-biphasic state, there appears to be a threshold below which EI is not detectable (Fig. 4*C*,*D*).

While qualitatively in both healthy and PD conditions cortical stimulation evoked an early excitation but in PD condition (both biphasic and triphasic) the duration and amplitude of early excitation were higher than that of in the healthy condition. This was because dopamine depletion amplified the excitation of SNr neurons through the hyperdirect pathway. Moreover, in PD condition when we could generate triphasic response pattern, the duration and amplitude of early inhibition (i.e., EI) were much smaller than that observed in the healthy condition (early inhibition was completely absent in the biphasic responses). Finally, the late excitation phase (LE) of the triphasic response was longer in PD condition than in the healthy condition. The details of further differences in transient response properties are provided in the Table 11. The trend of the features in normal and PD conditions is consistent with the experimental data (Ozaki et al., 2017; Sano and Nambu, 2019; Chiken et al., 2021; Wahyu et al., 2021).

**Table 11:** Features of the transient response of the SNr neurons

	Normal (triphasic)	PD-biphasic	PD-triphasic
Early excitation (EE)			
Latency (ms)	**7.0 ± 0**, *7.0 ± 0*	**7.0 ± 0**, *6.92 ± 0.56*	**7.0 ± 0**, *6.99 ± 0.10*
Duration (ms)	**4.0 ± 0**, *4.0 ± 0*	**21.98 ± 0.14**, *21.82 ± 0.74*	**5.0 ± 0**, *4.98 ± 0.20*
Deviation of peak amplitude from the baseline ( $H_{p}-H_{bas}$)	**62.07 ± 1.28**, *61.96 ± 2.4*	**70.77 ± 1.32**, *70.76 ± 2.42*	**169.95 ± 2.32**, *169.87 ± 4.49*
Early inhibition (EI)		*ND	
Latency (ms)	**11.0 ± 0**, *11.0 ± 0*		**12.0 ± 0**, *11.97 ± 0.17*
Duration (ms)	**6.0± 0.0**, *5.99 ± 0.10*		**4.0 ± 0.0**, *4.03 ± 0.17*
Deviation of peak amplitude from the baseline ( $H_{p}-H_{bas}$)	**−29.74 ± 0.24**, *−29.88 ± 0.48*		**−13.23 ± 0.40**, *−13.43 ± 0.70*
Late excitation (LE)		##	
Latency (ms)	**17.0 ± 0.0**, *16.99 ± 0.10*		**16.0 ± 0.0**, *16.0 ± 0.0*
Duration (ms)	**9.3 ± 0.59**, *9.16 ± 0.74*		**12.0 ± 0.0**, *12.0 ± 0.0*
Deviation of peak amplitude from the baseline ( $H_{p}-H_{bas}$)	**137.82 ± 1.44**, *138.08 ± 2.41*		**145.94 ± 1.39**, *146.01 ± 2.82*
Late inhibition (LI)			
Latency (ms)	**26.3 ± 0.59**, *26.15 ± 0.73*	**28.98 ± 0.14**, *28.74 ± 0.44*	**28.0 ± 0**, *28.0 ± 0*
Duration (ms)	**10.61 ± 1.17**, *10.65 ± 1.42*	**34.45 ± 0.5**, *34.77 ± 0.73*	**31.16 ± 0.36**, *31.40 ± 0.49*
Deviation of peak amplitude from the baseline ( $H_{p}-H_{bas}$)	**−6.57 ± 0.48**, *−7.31 ± 0.94*	**−24.26 ± 0.22**, *−24.48 ± 0.39*	**−19.79 ± 0.27**, *−20.10 ± 0.52*

Here, the variations in the features were obtained using multiple observations (100 in number) of the simulation output. In each observation, 50% and 20% of SNr neurons were randomly chosen whose results are shown in bold and italic, respectively. The format of the result is mean ± std. It can be seen that when 20% of neurons are used to compute the statistics, then the SD (std) increases, as expected because of the reduced number of neurons. Such a statistics is useful to study the variations that the population neuron brings into the network. ##the statistics for EE in PD-biphasic are given for the complete excitatory response comprising of both EE and LE. In this case, the EI was not detectable using statistical test, hence the two excitations (EE and LE) were merged during computation of the parameters. Here, the deviation of peak amplitude (*H_p_*) was measurable with respect to baseline (*H_bas_*). *ND denotes that the zone was not detected using significance test (see Materials and Methods, Data analysis).

These results suggest that dopamine depletion primarily affected the EI and LE zones. On one hand dopamine depletion reduced excitability of D1-SPN (Gruber et al., 2003) and reduced basal firing in D1-SPN while increasing in firing rate of D2-SPNs. Therefore, the direct pathway was weakened and resulted in reduced EI in the SNr. On the other hand the basal firing rate of GPe-TI was reduced and GPe-TA was increased (Mallet et al., 2008) because of the strengthening of the striato-pallidal pathway. This resulted in prolonged “LE” zone.

### STN evoked transient response in the SNr

To separate the contribution of direct and hyperdirect pathways we measured the SNr response when only STN was stimulated (Fig. 3, bottom row, green trace). In healthy state, consistent with experimental data (Maurice et al., 2003) and a previous modeling study (Lindahl et al., 2013), STN stimulation alone generated a triphasic response in the SNr; however, there were notable differences: the EI zone was weaker, LE zone was both weaker and delayed and, LI zone was absent. In this type of stimulation codition, STN to SNr connections shaped the EE zone, and STN↔GPe-TI connections shaped the EI zone and the LE zone. Because cortical inputs to the striatum promote inhibition in the GPe-TI and SNr, their removal made the EI and LE zone much weaker and diminished the LI zone.

In PD condition, STN stimulation induced a transient response with only EE zone (essentially, EE and LE zones observed in a healthy state were merged into a single excitatory zone). The magnitude of the EE zone was much higher in the PD triphasic configuration as compared with the normal state because of the stronger hyperdirect pathway. These results confirm that in healthy state the hyperdirect pathway shapes the EE zone, and suggest that the contribution of the striatal activity (direct pathways) is in increasing the magnitude while decreasing the duration of the EI and LE zones.

### Effect of the strength of cortical stimulation on the transient response

**Figure 5.**
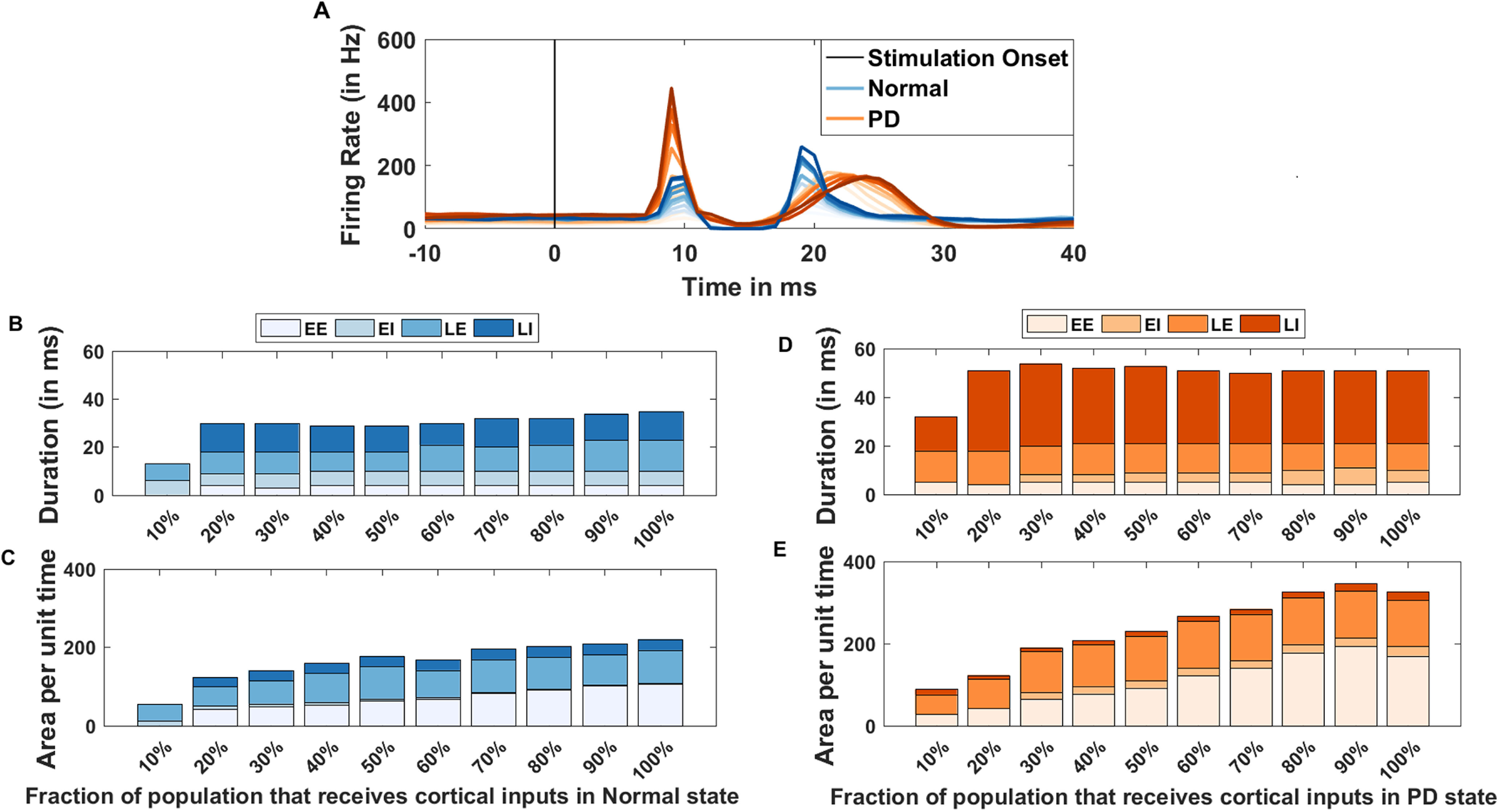
Effect of strength of cortical stimulation on BG transient response shape. To vary the strength of cortical stimulation we varied the fraction of striatal and STN populations that received cortical inputs from 10% to 100%. ***A***, Average transient response (100 trials) in SNr in normal (blue color) and PD state (orange color). Lighter (darker) color-shades indicate smaller (larger) size of stimulated population. Note that in PD condition, even the strongest cortical input failed to elicit a response similar to that seen in healthy state. ***B***, Changes in the duration of the four zones of the transient response in normal state. ***C***, Changes in the area per unit time (area/time) of the four zones of the transient response in normal state. ***D***, Same as in ***B*** but for PD state when the network responded with a triphasic response. ***E***, Same as in ***C*** but for PD state when the network responded with a triphasic response. Note missing colors in a given bar implies that we could not detect the corresponding zone.

The aforementioned transient responses were measured by stimulating 50% of the striatal and STN population. Next, we asked whether differences in the shape of the triphasic response observed in PD and healthy conditions could be reduced by stimulating more neurons. To this end, we systematically increased the number of striatal and STN neurons that received cortical stimulation (to mimic the strength of cortical stimulation). To quantify the changes in the shape of the triphasic responses we measured the duration and area per unit time of the four zones in healthy (Fig. 5*B*,*C*) and PD condition (Fig. 5*D*,*E*).

We found that in both healthy and PD conditions, the amplitude of the four zones are monotonically increased and saturated at a maximum value (Fig. 5*A*). On the other hand, area per unit time of the excitatory zones monotonically increased. Thus, an increase in the stimulus strength increased both excitation and induced stronger inhibition in healthy state. However, in healthy state a very weak cortical stimulation (10%) failed to elicit a detectable EE response (Fig. 5*B*,*C*) but in PD condition (Fig. 5*D*,*E*), the same weak stimulation elicited a strong EE response, again indicated the strengthening of the hyperdirect pathway in low-dopamine state.

Overall, these results show that even with the strongest stimulation in PD condition, we could not reproduce the transient response properties observed in healthy state even with the weakest cortical stimulation. This suggests that the differences in the transient response are not simply because of the altered cortico-BG projections but are primarily because of the altered connectivity within the BG.

### Effect of change in synaptic connections on cortical evoked transient response in SNr

Above, we demonstrated the transient response pattern for a specific combination of synaptic strengths. The total space of different synaptic parameters is 22-dimensional (Table 2), and therefore, it is not feasible to test the robustness of our results in a systematic manner by varying all the connection parameters. The structure of BG connectivity suggests that the triphasic response pattern is shaped by D1-SPN →SNr (early inhibition), GPe-TA ↔GPe-TI, STN ↔GPe-TI, and D2-SPN →GPe-TI (late excitation/inhibition) connectivity. Therefore, we individually varied these six connections and quantified the duration and area per unit time (area/time) of the four zones of the transient response. The minimum and maximum values of each of the synaptic weight (except D1-SPN→SNr synapses) corresponded to their values in L-dopa-induced dyskinesia (LID) and PD conditions, respectively (see Materials and Methods; Table 10). For the case of D1-SPN →SNr synapses minimum and maximum values corresponded to PD condition and LID, respectively (see Table 10). To mimic low-dopamine and high-dopamine states, we varied the connection strengths in the following manner. Let’s consider that the synaptic weight in normal condition is *v* and in PD condition is *mv* (where the scaling factor *m* was derived from Table 10). To mimic high-dopamine (greenish colors) we reduced *v* to *v*/*m* in three steps. Similarly, to mimic low-dopamine (reddish colors) we increased *v* to *mv* in three steps. Thus, the simulations were done for seven different configurations of synaptic strengths including the normal.

**Figure 6.**
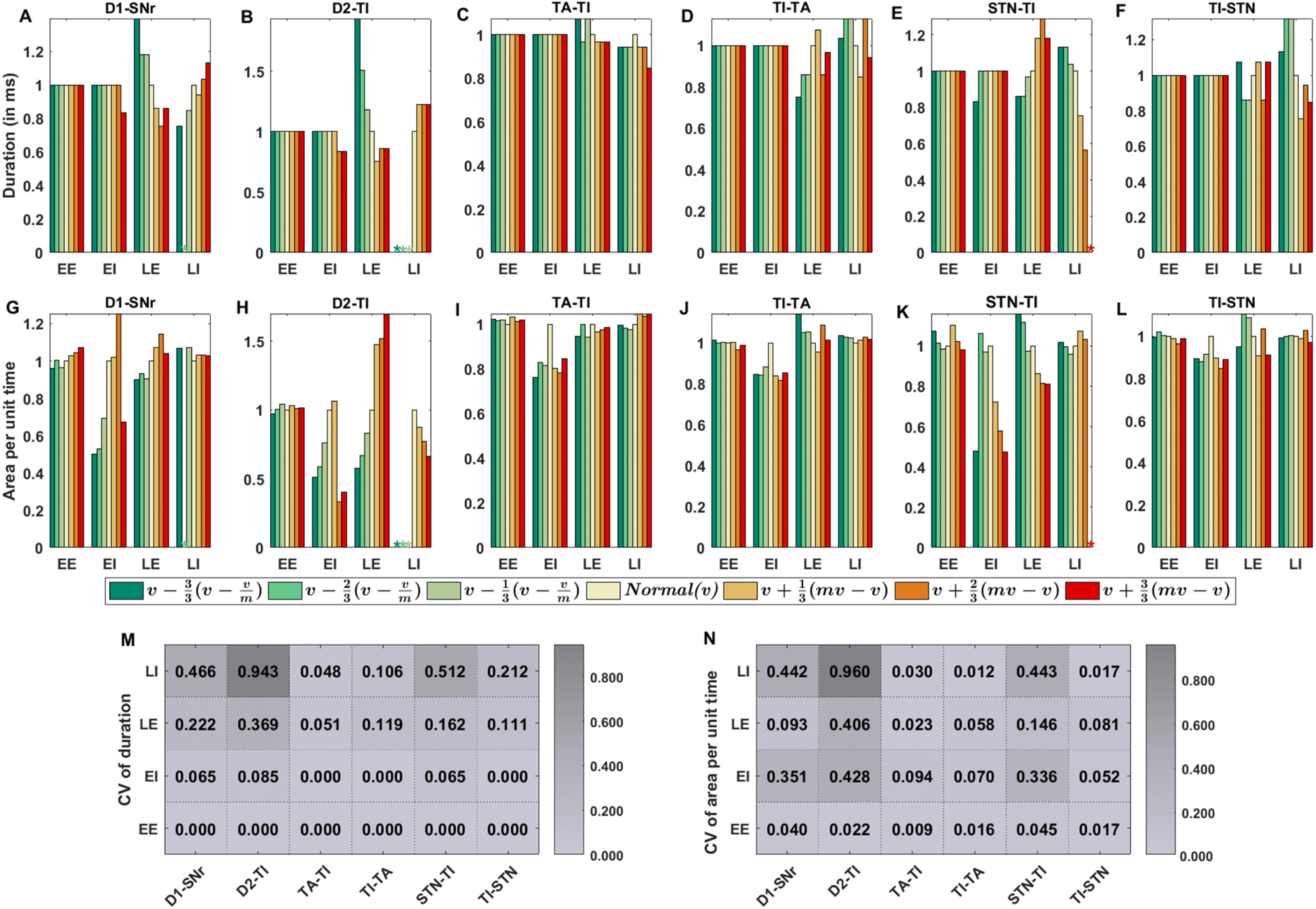
Effect of synaptic weight changes of 6 different connections. We varied the strength of D1-SPN →SNr (D1-SNr), D2-SPN →GPe-TI (D2-TI), GPe-TA →GPe-TI (TA-TI), GPe-TI →GPe-TA (TI-TA), STN →GPe-TI (STN-TI), and GPe-TI →STN (TI-STN) from their baseline values in a normal condition. We changed the connection strength from *v*/*m* to *mv* in seven steps (for more information, see Materials and Methods). Cortical input was given to 50% of the striatal and STN populations. ***A–F***, Change in duration of the four zones as the strength of one of the connections was increased (3 values) or decreased (3 values) to mimic low and high dopamine states. The duration of each zone was normalized with respect to duration in the normal condition for that particular zone (beige colored bars). ***G–L***, Same as in panels ***A–F*** but for area per unit time for each of the four zones. LI: late inhibition, LE: late excitation, EI: early inhibition, EE: early excitation; ⋆: not detected. ***M***, CV of the duration of the four zones duration as a function of the six synaptic connections (computed from ***A–F***). High value of CV means that changes in the particular connection result in higher changes (increase/decrease) in the duration of a zone. ***N***, Same as in panel ***M*** but for area per unit time.

We found that synaptic weight changes can affect the duration of the LE and LI zones, but not of EE and EI zones (Fig. 6*A–F*). By contrast, the area per unit time of all four zones was sensitive to synaptic weight changes (Fig. 6*G–L*). Our simulation experiments showed that mainly the connection between the D1-SPN →SNr (Fig. 6*A*,*G*), the STN →GPe-TI (Fig. 6*E*,*K*), and D2-SPN →GPe-TI (Fig. 6*B*,*H*) controlled the shape of the three zones namely EI, LE, and LI. To analyze the relative contribution of these connections, we calculated the coefficient of variation (CV) of duration (Fig. 6*M*) and area/time (Fig. 6*N*) of the four zones for each of the six connections we changed. Smaller the CV values, smaller is the influence of that connection on the transient response properties.

From this analysis the D2-SPN →GPe-TI connection emerged as the most crucial parameter in shaping the transient response in both low and high-dopamine conditions. For extreme values of D2-SPN →GPe-TI connection, area/time of late excitation was very high in low dopamine condition and late inhibition zone was completely absent in high dopamine state. Besides D2-SPN→GPe-TI connection, STN →GPe-TI connection was the second most important parameter as it affected the EI, LE, and in particular, the LI zones. Finally, it was somewhat surprising that the GPe-TI ↔GPe-TA and GPe-TI →STN connections did not affect the area/time of any of the zones (Fig. 6*I*,*J*,*L*).

### Effect of restoration of dopaminergic synaptic connection on the transient response in SNr

To get further insights into the network mechanisms underlying the aberrant transient response, we asked whether we could restore the healthy state of transient response by restoring specific connections to their healthy levels. In the previous section we showed that the D1-SPN →SNr, STN →GPe-TI, and D2-SPN →GPe-TI (Fig. 6*M*,*N*) have the strongest effect on the triphasic response. To further confirm their role in the generation of aberrant transient responses, we restore selected connections along the indirect pathway (i.e., D2-SPN →GPe-TI or STN ↔GPe-TI loop, or GPe-TA ↔GPe-TI).

To this end, first, we tuned the BG network in the PD state (Table 10) such that the SNr shows a triphasic response. Then restored the strength of D2-SPN →TI, STN ↔GPe-TI loop, GPe-TA ↔GPe-TI, and D1-SPN →SNr one by one. During the restoration of the synaptic connection between a pair of nuclei, (1) the synaptic weights and delays were made equal to normal, (2) the basal firing rates were made similar to the normal by changing the background firing rate or the background current, (3) basal firing of SNr was kept the same as that of the normal condition. To compare the triphasic response in healthy and PD states (with and without restoration of certain synaptic weights) we measured the distance between two network conditions (Eq. 5; see Materials and Methods, Data analysis).

**Figure 7.**
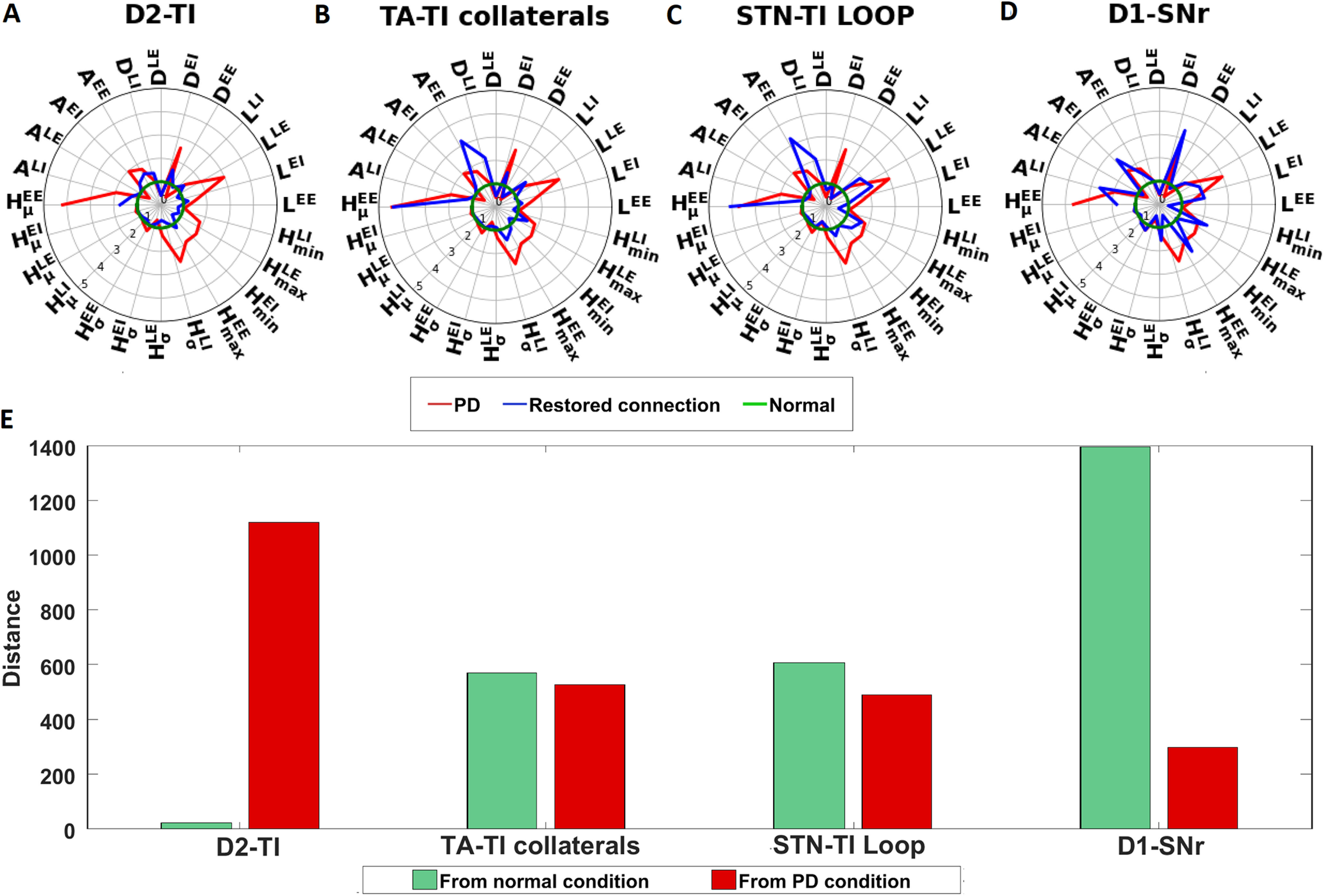
Effect of restoration of synaptic weights of D1-SPN →SNr (D1-SNr), D2-SPN →GPe-TI (D2-TI), STN ↔GPe-TI (STN-TI loop), and GPe-TA ↔GPe-TI connections after dopamine depletion. Cortical input was given to 50% of the striatal and STN neurons. ***A***, Comparison of zone wise feature values for test network condition with respect to the healthy and PD states. Here, the test network refers to a PD network (tuned to generate triphasic response) in which synaptic weight of D2-SPN →GPe-TI was only restored to their healthy value (restored connection, D2-TI). Feature values for healthy condition is represented as unit circle (shown in green). *L^EE^*, *D^EE^*, *A^EE^*, $H_{\mu }^{EE},\;H_{\sigma }^{EE}$, and $H_{max}^{EE}$ denote the latency, duration, area, mean of bin heights, SD of bin heights, and maximum of bin heights for EE zone, respectively (for feature description, see Materials and Methods, Data analysis). *L^EI^*, *D^EI^*, *A^EI^*, $H_{\mu }^{EI},\;H_{\sigma }^{EI}$, and $H_{min}^{EI}$ denote the latency, duration, area, mean of bin heights, SD of bin heights, and minimum of bin heights for EI zone, respectively. *L^LE^*, *D^LE^*, *A^LE^*, $H_{\mu }^{LE},\;H_{\sigma }^{LE}$, and $H_{max}^{LE}$ denote the latency, duration, area, mean of bin heights, SD of bin heights, and maximum of bin heights for LE zone, respectively. *L^LI^*, *D^LI^*, *A^LI^*, $H_{\mu }^{LI},\;H_{\sigma }^{LI}$, and $H_{min}^{LI}$ denote the latency, duration, area, mean of bin heights, SD of bin heights, and minimum of bin heights for LI zone, respectively. ***B***, Same as ***A*** but when GPe-TA ↔GPe-TI connections were restored (restored connection, TA-TI collaterals). ***C***, Same as ***A*** but when STN ↔GPe-TI loop were restored (restored connection, STN-TI loop). ***D***, Same as ***A*** but when D1-SPN →SNr was restored (restored connection, D1-SNr). ***E***, Distance computed for different test network conditions based on ***A–D*** from healthy and PD network state. The green bars indicate the distance (calculated using Eq. 5) between healthy network activity state and test network activity state, whereas red bars indicate the distance between PD network activity state and test network activity state. Here, the test network refers to a PD network (tuned to generate triphasic response) in which individual synaptic weights (mentioned on the *x*-axis) were restored to their healthy value.

We found that restoring the D2-SPN →GPe-TI alone is sufficient to bring the shape of the transient response close to the one observed in healthy state (Fig. 7*A*,*E*). However, by restoring the GPe-TI ↔GPe-TA or STN ↔GPe-TI connections, we only restored the shape of EI and LE zone but not that of the EE and LI zone (Fig. 7*B*,*C*). By contrast, restoration of the D1-SPN →SNr synaptic connection made the network activities different from as in both PD and healthy conditions (Fig. 7*D*,*E*). This is because in PD condition, in addition to the weakening of D1-SPN to SNr synapses, cortical inputs to D1-SPN were also weakened (Lindahl and Kotaleski, 2016), and therefore, cortical stimulation only evokes a weak response in D1-SPN. Thus, although restoration of the D2-SPN →GPe-TI or STN ↔GPe-TI makes the transient response in PD condition more similar to the healthy condition, the early inhibitory phase is not restored.

### Ongoing spontaneous activity of BG network

In the steady states, the β band oscillations and synchrony within and between different BG subnuclei during stimulus free ongoing spontaneous activity are two prominent hallmarks of PD condition *in vivo* (Brown et al., 2001; Mallet et al., 2006, 2008). Therefore, next we tested whether the network parameters we used to generate the aberrant triphasic and biphasic responses could also induce β band oscillations. To this end, we tuned the BG network in PD condition when it showed either triphasic or biphasic transient response and measured the oscillations and synchrony in the ongoing (stimulus-free or spontaneous) activity.

**Figure 8.**
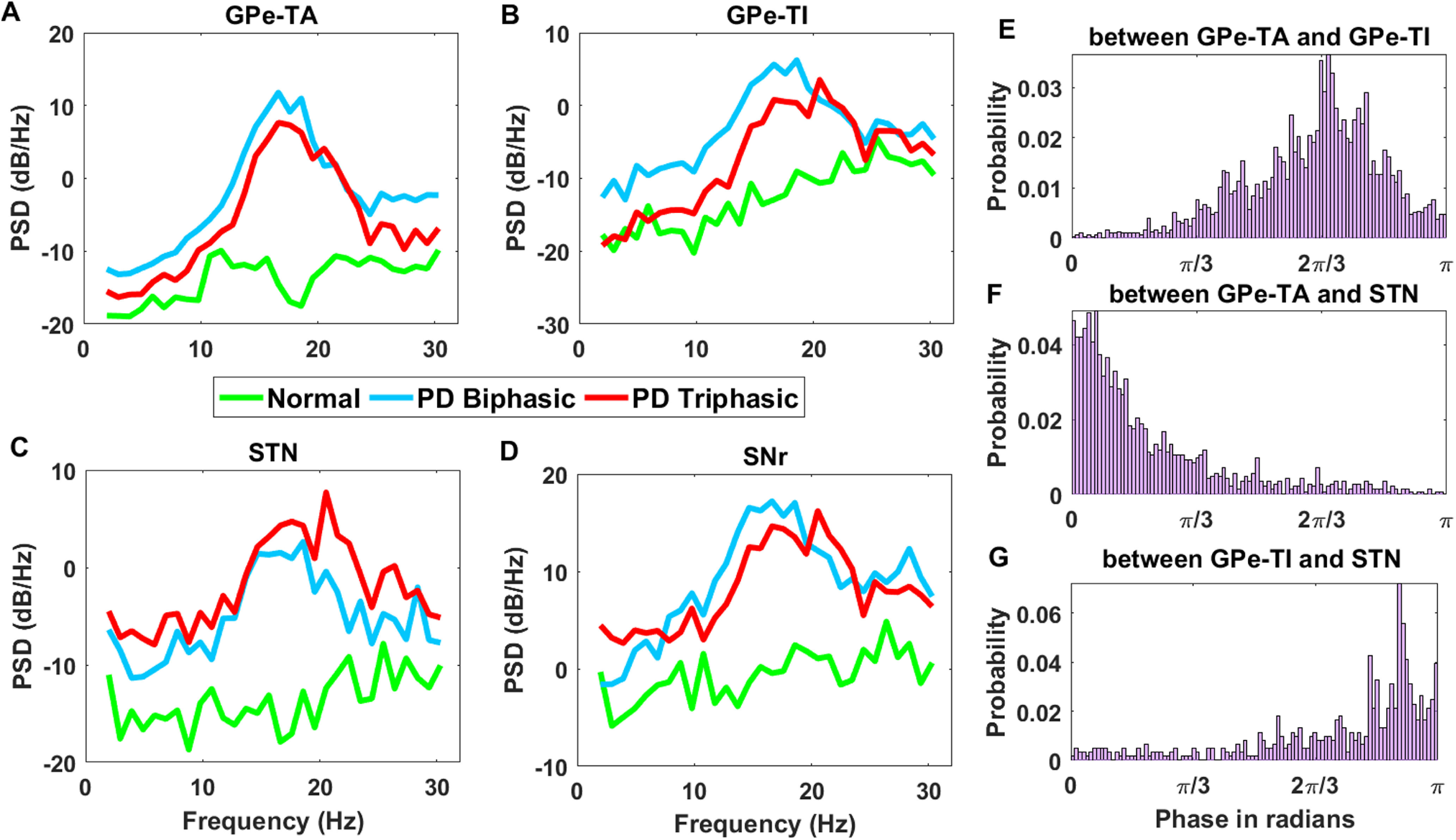
β band oscillations in the ongoing activity of the BG. ***A***, Spectrum of the GPe-TA activity in healthy (green), PD-triphasic (red), and PD-biphasic (brown) response conditions. ***B***, Same as in panel ***A*** but for GPe-TI. ***C***, Same as in panel ***A*** but for STN. ***D***, Same as in panel ***A*** but for SNr. ***E***, Phase relation between GPe-TA and GPe-TI shown in the range of 0 and *π* (in radians). ***F***, Same as ***E*** but for the phase relation between GPe-TA and STN. ***G***, Same as ***E*** but for the phase relation between GPe-TI and STN. The phase difference between GPe-TA and GPe-TI attains a peak around $\frac{2\pi }{3}$ in the phase histogram shown in ***E***. The in-phase relation between GPe-TA-STN and approximate anti-phase relation between GPe-TI-STN can be seen in ***F***, ***G***, respectively.

We found that indeed the same set of parameters that generated aberrant transient responses were sufficient to elicit clear β band oscillations in both the biphasic and triphasic response modes (Fig. 8*A–D*). Next, we measured the phase relationship between different subnuclei of the BG. Mallet et al. (2008) reported that there exists an in-phase relationship between activities of GPe-TA and STN neurons and anti-phase relationship between GPe-TA and GPe-TI neurons. In our model the phase relationships between GPe-TA and GPe-TI, GPe-TA and STN, GPe-TI and STN (Fig. 8*E–G*) were similar to that observed in experimental data. Thus, these results suggest that similar changes in the network connection could underlie the aberrant transient response and ongoing activity in PD conditions as well.

### Effect of striato-pallidal and pallido-subthalamic pathways on the β oscillations

While β band oscillations are a clear neural signature of PD, the mechanisms underlying the emergence of these oscillations are still debated. Both experimental data (Plenz and Kital, 1999; Hammond et al., 2007; Tachibana et al., 2011; de la Crompe et al., 2020) and computational models (Kumar et al., 2011; Holgado et al., 2010; Tachibana et al., 2011; Pavlides et al., 2015; Corbit et al., 2016; Bahuguna et al., 2020) have implicated essentially all the various network interactions in generating oscillations. Here, we have developed the BG model primarily to understand the transient response and found that the same model can also generate β band oscillations. Thus, we have a more constrained model of the BG than used previously, and this could help us narrow down on the key determinants of oscillations.

Based on our simulations and available experimental data (de la Crompe et al., 2020) GPe has emerged as a key network necessary to induce β band oscillations. However, it remains unclear which of its input and output connections are more crucial to generate oscillatory activity. Therefore, to quantify the relative contribution of GPe connectivity we computationally followed the path of lesion experiments usually done *in vivo* studies. To do this, we either removed striatal input to GPe-TI neurons, or GPe feedback to the striatal FSIs, or GPe-STN interactions. All these perturbations were performed in two different BG networks which showed biphasic or triphasic response in PD condition.

**Figure 9.**
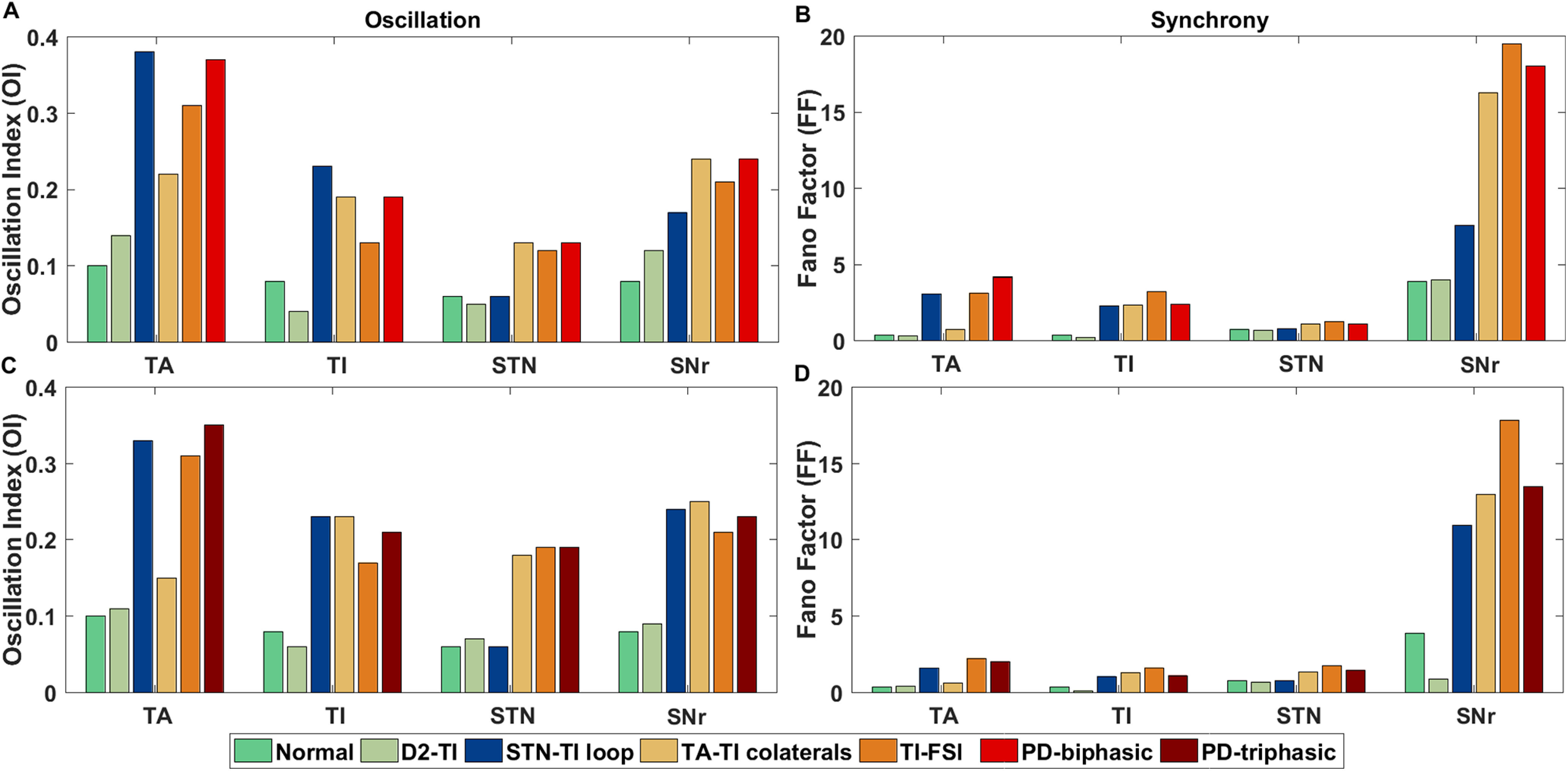
Comparison of relative changes in oscillation and synchrony when synaptic connections between D2-SPN →GPe-TI (D2-TI), STN ↔GPe-TI (STN-TI loop), GPe-TI ↔GPe-TA (TA-TI collaterals), and GPe-TI →FSI (TI-FSI) were disconnected. ***A***, Oscillation Index for GPe-TA, GPe-TI, STN, and SNr with normal, PD-biphasic states and with lesioned networks. ***B***, Fano factor for GPe-TA, GPe-TI, STN, and SNr with normal, PD-biphasic states and with lesioned networks. ***C***, Same as ***A*** while comparing with the PD-triphasic state. ***D***, Same as ***B*** while comparing with the PD-triphasic state.

In both PD conditions (biphasic response and triphasic response), removal of D2-SPN input to GPe-TI neurons reduced the oscillations and synchrony GPe-TA, GPe-TI, STN, and SNr neurons nearly to a level observed in healthy state (Fig. 9, pale green bars). The convergence of a relatively large numbers of D2-SPNs onto a smaller set of the GPe-TI neurons greatly influenced the patterning of GPe-TI activity (Kovaleski et al., 2020). This supports the hypothesis that increase in D2-SPN activity in dopamine depleted state is responsible for unleashing oscillations in the BG (Mallet et al., 2006; Kumar et al., 2011; Sharott et al., 2017). Moreover, recent experiments also suggest that D2-SPN inputs control oscillations in the GPe-TI population (de la Crompe et al., 2020).

By contrast, removal of GPe feedback to striatal FSIs (Fig. 9, orange bars) did not affect oscillations or synchrony considerably. The effect of interactions within and between GPe-TA and GPe-TI neurons was dependent on the state of the network: removal of these connections (Fig. 9, yellow bars) reduced oscillations and synchrony of GPe-TA, GPe-TI, STN, and SNr neurons by a larger amount when the BG was tuned to exhibit triphasic response in PD condition (Fig. 9*C*,*D*, yellow bars). However, even after removal of collateral within the GPe neurons, both oscillations and synchrony were much higher than that observed in a healthy state.

Surprisingly, removal of STN ↔GPe-TI connections did not affect the oscillation, regardless of the type of transient responses the network showed in PD condition (Fig. 9, blue bars). These findings imply that following dopamine loss, the abnormal patterning of GPe-TI by stronger striato-pallidal connection was opposed by STN-GPe transmission and thus, removal or inhibition of the STN will also not have any effect on the oscillations (Kovaleski et al., 2020). This is consistent with the recent findings by de la Crompe et al. (2020), who showed that optogenetic inhibition of STN does not quench oscillations (see also Gradinaru et al., 2009). Although experimental evidences suggest that the oscillatory dynamics in SNr in dopamine depleted state could be induced through either stronger D2-SPN →GPe-TI (Gradinaru et al., 2009; de la Crompe et al., 2020) or STN ↔GPe-TI loop (Holgado et al., 2010; Pavlides et al., 2015); however, in our model, the removal of the D2-SPN →GPe-TI connection is sufficient to suppress the oscillation in the SNr. The results emphasize the role the indirect pathway as opposed to the hyperdirect pathway in the manifestation of aberrant activity in PD.

### Diversity of transient responses

As noted earlier, our BG network model is homogeneous and therefore, we could either generate biphasic or triphasic shaped transient response in the network. This approach however, allowed us to identify the key network interactions that are involved in changing the response shape from biphasic to triphasic (i.e., D2-SPN →GPe-TI, D1-SPN →SNr, and GPe-TI →STN). An inhomogeneous change in these connections could be one of the reasons for the observed diversity of transient responses in *in vivo*. However, oscillations in the ongoing activity could also contribute to the diversity of transient responses because the shape of transient response may depend on the oscillation phase at which cortical stimulation was delivered. In fact, recent experimental data suggests that when β band oscillations are weak or absent in PD, transient responses variability is reduced (Chiken et al., 2021).

To test this hypothesis, we tuned the network in a PD state in which it responded with a biphasic shape (Table 10) and delivered the stimulus at different phases of oscillations. There were 48 trials of each cortical stimulus given at a specific phase of the SNr oscillation. We pooled the data for each of such 48 trials and observed various responses namely, “EE-EI-LE,” “EE-EI,” “EI-LE,” “EE-LE,” “EE,” and “LE.” Here, “EE-EI-LE” denotes a triphasic response which is observed mainly in healthy state.

**Figure 10.**
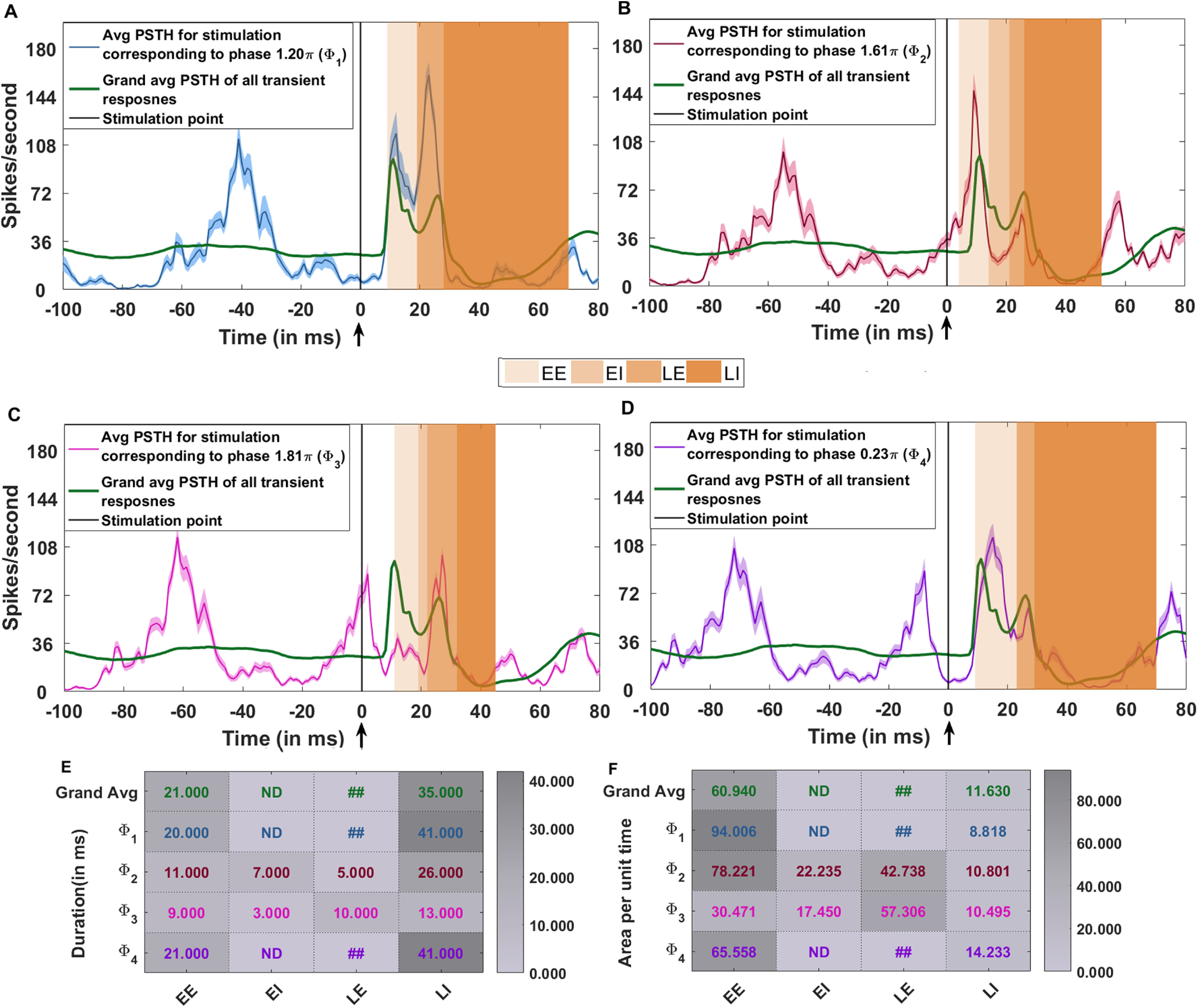
Diversity of transient response may depend on the phase of cortical stimulation. The network was tuned to operate in a PD state in which transient inputs result in a biphasic response in the SNr. Cortical input was given to 50% of the striatal and STN neurons. ***A***, blue trace, Average transient response (average across 48 trials) when the stimulation arrived at trough of the β oscillations (Φ_1_ = 1.20*π*). It can be seen that during the stimulation, SNr is close to the trough of oscillation. The shaded areas above/below the transient response (blue line) denote the 95% confidence interval. Green trace, The grand average (grand average) of the transient response averaged across all trials and stimulation phases. The corresponding transient response (shown in blue) with only EE and LE zones. ***B***, Same as panel ***A***; however, the stimulation arrived at a phase Φ_2_ = 1.61*π*. The corresponding transient response (shown in dark red) is triphasic (EE-EI-LE). ***C***, Same as ***A***; however, the stimulation arrived at a phase Φ_3_ = 1.81*π*, i.e., very close to the peak of SNr oscillation. The corresponding transient response (shown in magenta) is triphasic (EE-EI-LE); however, the EI zone is very weak. ***D***, Same as ***A***; however, the stimulation arrived at a phase Φ_4_ = 0.23*π*, i.e., on the falling edge close to the trough of SNr oscillation. The corresponding transient response (shown in violet) is biphasic (EE-LE). ***E***, Variation in the zone wise duration of the transient response for the stimulation corresponding to phase $\Phi_{1},\;\Phi_{2},\;\Phi_{3}$, and Φ_4_. ***F***, Variation in the zone wise area per unit time (area/time) feature of the transient response for the stimulation corresponding to phase $\Phi_{1},\;\Phi_{2},\;\Phi_{3}$, and Φ_4_. *^##^*The duration and area/time corresponding to phase $\Phi_{1},\;\Phi_{4}$ and for the grand average are given for the complete excitatory response comprising of both EE and LE zones. In these cases, the EI was not detectable using statistical test, hence the two excitations (EE and LE) were merged during computation of the parameters. ND denotes that the zone was not detected using significance test (see Materials and Methods, Data analysis).

This variation was primarily because of the differences in phase of the oscillation at which cortical stimulation was delivered. We found that when the input arrived at the trough (1.20*π*; Fig. 10*A*) or during the falling edge of the SNr β oscillations (0.23*π*; Fig. 10*D*), SNr responded with a biphasic transient response. The magnitude of the LE zone was stronger when the input arrived at the trough instead of the falling phase of the β oscillations (Fig. 10*A*,*D*).

By contrast, when the stimulation arrived during the rising phase, it resulted in triphasic responses in SNr (Fig. 10*B*,*C*), although the strength (area/time) and duration of the EE, EI, and LE varied with the actual phase of the stimulation (Fig. 10*E*,*F*). It is important to note that the triphasic response observed for some stimulation phases was still quantitatively different from that triphasic response seen in a healthy state.

In order to characterize the contribution of different BG nuclei to the transient response in healthy and PD conditions, we varied the strength of several connections in BG (e.g., see Figs. 5, 6). We pooled all those simulations together, where strengths of the synaptic connection were varied according to Figure 6, and estimated the variability of the transient responses. The rationale to do this was that each network simulation with different connection strength may represent a different SNr/GPi region or animal where the transient response was recorded. Indeed, such pooling of the data resulted in a high heterogeneity in the transient responses in both healthy and PD conditions (see Table 12), which closely matched with the experimental data.

**Table 12:** Features of the transient response of the SNr neurons, same as **Table 11; however, by pooling synaptic weights corresponding to Figure 6**

	Normal (triphasic)	PD-biphasic	PD-triphasic
Early excitation (EE)			
Latency (ms)	**7.0 ± 0**, *6.93 ± 0.50*	**7.0 ± 0**, *7.0 ± 0*	**7.0 ± 0**, *7.0 ± 0*
Duration (ms)	**3.0 ± 0**, *4.04 ± 0.43*	**23.0 ± 0.79**, *21.01 ± 0.08*	**3.69 ± 0.46**, *4.03 ± 0.18*
Deviation of peak amplitude from the baseline ( $H_{p}-H_{bas}$)	**38.02 ± 2.76**, *63.30 ± 4.39*	**75.84 ± 12.53**, *67.14 ± 1.65*	**153.63 ± 6.43**, *159.96 ± 3.67*
Early inhibition (EI)		*ND	
Latency (ms)	**10.0 ± 0**, *11.0 ± 0*		**10.69 ± 0.46**, *11.03 ± 0.18*
Duration (ms)	**5.56 ± 0.49**, *6.0 ± 0.0*		**3.66 ± 0.77**, *4.96 ± 0.18*
Deviation of peak amplitude from the baseline ( $H_{p}-H_{bas}$)	**−29.18 ± 0.54**, *−29.64 ± 0.44*		**−11.7 ± 1.73**, *−15.16 ± 0.34*
Late excitation (LE)		##	
Latency (ms)	**15.56 ± 0.49**, *16.94 ± 0.74*		**14.35 ± 0.47**, *16.0 ± 0.0*
Duration (ms)	**7.66 ± 0.51**, *9.08 ± 0.49*		**14.21 ± 0.71**, *12.0 ± 0.0*
Deviation of peak amplitude from the baseline ( $H_{p}-H_{bas}$)	**156.72 ± 16.24**, *136.91 ± 1.57*		**139.45 ± 2.91**, *147.74 ± 1.30*
Late inhibition (LI)			
Latency (ms)	**23.22 ± 0.42**, *26.03 ± 0.54*	**30.0 ± 0.79**, *28.00 ± 0.08*	**28.57 ± 0.49**, *28.0 ± 0.0*
Duration (ms)	**13.17 ± 0.96**, *10.53 ± 0.86*	**44.09 ± 9.35**, *37.24 ± 0.59*	**31.8 ± 1.11**, *31.99 ± 0.09*
Deviation of peak amplitude from the baseline ( $H_{p}-H_{bas}$)	**−11.81 ± 1.64**, *−5.11 ± 0.51*	**−16.6 ± 1.72**, *−20.65 ± 0.30*	**−19.55 ± 1.31**, *−20.83 ± 0.34*

Here, the variations in the features in normal state were obtained by simulating the network with the range of synaptic weights of a particular connection between (*v* - $\frac{\left(v-v/m\right)}{3}$) and (*v* + $\frac{\left(mv-v\right)}{3}$). Similarly, the variations in the features in the PD conditions (PD-biphasic and PD-triphasic) were obtained by simulating the network with the range of synaptic weights of a particular connection between (*v* + $\frac{\left(mv-v\right)}{3}$) and (*v* + $\frac{3(mv-v)}{3}$) (*mv*, i.e., weight in PD condition). These were done by considering six types of synaptic connections corresponding to Figure 6.

**Table 13:** Comparison of features corresponding to the shape of transient response in the SNr before and after increasing the excitability of D2-SPNs in PD-biphasic and PD-triphasic states

	PD-biphasic (same as Table 11)	PD-biphasic	PD-triphasic (same as Table 11)	PD-triphasic
EE				
Latency (ms)	**7.0 ± 0**, *6.92 ± 0.56*	**7.0 ± 0**, *7.0 ± 0.0*	**7.0 ± 0**, *6.99 ± 0.10*	**7.0 ± 0**, *7.0 ± 0.0*
Duration (ms)	**21.98 ± 0.14**, *21.82 ± 0.74*	**22.18 ± 0.38**, *22.21 ± 0.43*	**5.0 ± 0**, *4.98 ± 0.20*	**4.05 ± 0.21**, *4.2 ± 0.40*
$H_{p}-H_{bas}$	**70.77 ± 1.32**, *70.76 ± 2.42*	**76.19 ± 1.24**, *76.6 ± 2.34*	**169.95 ± 2.32**, *169.87 ± 4.49*	**165.9 ± 2.19**, *165.52 ± 4.59*
EI	*ND	*ND		
Latency (ms)			**12.0 ± 0**, *11.97 ± 0.17*	**11.05 ± 0.21**, *11.2 ± 0.4*
Duration (ms)			**4.0 ± 0**, *4.03 ± 0.17*	**4.95 ± 0.21**, *4.8 ± 0.4*
$H_{p}-H_{bas}$			**−13.23 ± 0.40**, *−13.43 ± 0.70*	**−15.55 ± 0.44**, *−15.7 ± 0.74*
LE	##	##		
Latency (ms)			**16.0 ± 0**, *16.0 ± 0*	**16.0 ± 0**, *16.0 ± 0*
Duration (ms)			**12.0 ± 0**, *12.0 ± 0*	**12.0 ± 0**, *12.0 ± 0*
$H_{p}-H_{bas}$			**145.94 ± 1.39**, *146.01 ± 2.82*	**149.65 ± 1.23**, *150.27 ± 2.59*
LI				
Latency (ms)	**28.98 ± 0.14**, *28.74 ± 0.44*	**29.18 ± 0.38**, *29.21 ± 0.43*	**28.0 ± 0**, *28.0 ± 0*	**28.0 ± 0**, *28.0 ± 0*
Duration (ms)	**34.45 ± 0.5**, *34.77 ± 0.73*	**33.3 ± 0.61**, *33.34 ± 0.6*	**31.16 ± 0.36**, *31.40 ± 0.49*	**30.6 ± 0.46**, *30.6 ± 0.55*
$H_{p}-H_{bas}$	**−24.26 ± 0.22**, *−24.28 ± 0.39*	**−24.98 ± 0.28**, *−25.14 ± 0.41*	**−19.79 ± 0.27**, *−20.1 ± 0.52*	**−22.59 ± 0.29**, *−22.52 ± 0.53*

Column 1 and column 3 are taken from Table 11. Here, the variations in the features were obtained using multiple observations (100 in number) of the simulation output. In each observation, 50% and 20% of SNr neurons were randomly chosen whose results are shown in bold and italic, respectively. ##: Statistics for EE in PD-biphasic are given for the complete excitatory response comprising of both EE and LE. In this case, the EI was not detectable using statistical test, hence the two excitations (EE and LE) were merged during computation of the parameters. Here, $H_{p}-H_{bas}$ denotes the deviation of peak amplitude (*H_p_*) with respect to baseline (*H_bas_*). *ND denotes that the zone was not detected using significance test (see Materials and Methods, Data analysis).

These results while they do not explain the full diversity of the responses observed in *in vivo*, they show that the oscillation phase as well as diversity of synaptic connectivity are important variables in determining the shape of the response.

## Discussion

Here, we have studied how the changes induced by low-dopamine affect both transient response (induced by cortical stimulation) as well as the ongoing spontaneous activity state of the BG network. Typically, a transient stimulation of the cortex results in a triphasic response in the SNr/GPi (the output of the BG). The shape of the response is impaired in chronic low-dopamine conditions such as PD. The different zones of the transient response can be associated with different aspects of initiation of voluntary movements. For instance, it has been hypothesized that EE zone resets the cortical activity, EI zone allows for the execution of movements and LE zone stops the movement (Nambu et al., 2002; Chiken et al., 2021). A weaker or completely absent EI zone in PD is thought to be related to akinesia. Indeed, L-dopa treatment or local inhibition of the STN both of which restore the EI zone also ameliorate motor deficits in PD (Chiken et al., 2021). The triphasic response in the SNr/GPi is usually explained by difference in the relative timing of the hyperdirect, direct and indirect pathways of the BG which converge in the SNr/GPi.

Here, we show that changes in the shape of the transient response in PD state involve not only changes in the feed-forward connections between different subnuclei of the BG (D1-SPN →SNr) but also by interactions between STN and GPe (GPe-TI ↔STN; Fig. 6*K*) and to some extent by GPe-TA ↔GPe-TI (Fig. 7). Moreover, we show that same changes in the BG network (both synaptic and neuronal excitability) may underlie the impairment of transient response and emergence of induced population level oscillations and synchrony in the BG.

In the PD condition, neurons either show biphasic or triphasic transient responses (Sano and Nambu, 2019; Chiken et al., 2021; Wahyu et al., 2021); the latter is, however, quantitatively different from the triphasic response observed in healthy state. In our model, the aberrant biphasic response in PD condition appeared as we changed the parameters to a low-dopamine state (according to the model by Lindahl and Kotaleski, 2016). However, to obtain a triphasic response, we needed to reduce D2-SPN →GPe-TI, increase D1-SPN →SNr, and reduce GPe-TI →STN connections (see Table 10). This suggests that dopamine effects are not homogeneous within and between different subnuclei of BG. To restore a healthy state, it is important to experimentally characterize the heterogeneity of dopamine action. The diversity of dopamine action and phase of oscillations at which stimulation was delivered, together could explain the observed diversity of transient responses in *in vivo*.

Previously, Blenkinsop et al. (2017) suggested that in a healthy state, biphasic and triphasic responses in the SNr arise because of interactions among functionally segregated channels of competing inputs with different strengths. In a BG model with functionally segregated channels, local inhibition within the GPe and excitation from a small number of highly active STN neurons (presumably because of stronger cortical inputs) are responsible for emergence of LE zone, rendering a response biphasic or triphasic (Blenkinsop et al., 2017). Here, we have used a BG model without functionally segregated channels. Our results suggest that diversity of synaptic strengths within and between BG nuclei could give rise to some neurons responding in a triphasic and others in a biphasic manner. Consistent with the model by Blenkinsop et al. (2017), in our model, the magnitude of LE zone can be controlled by the strength of cortical stimulation (Fig. 5). Our work points out a strong influence of the indirect pathway (D2-SPN to GPe-TI) in controlling the shape of the transient response both in normal and PD condition. This observation is also consistent with the proposal of Blenkinsop et al. (2017).

Although we managed to generate a triphasic response in PD condition, it was quantitatively different from the one observed in a healthy state. The differences were most clearly seen in the late excitation which was lasted longer in PD condition as compared with a healthy state. Moreover, these differences in the triphasic response could not be compensated by increasing the magnitude of the cortical stimulation, suggesting that impaired transient response also entails impaired recurrent interactions within and between BG subnuclei.

Here, we assumed that the GPe to SNr and STN to SNr synapses are static. However, experimental data suggests that synapses between GPe to SNr show short-term depression (Connelly et al., 2010). Lindahl et al. (2013) has argued that when GPe to SNr synapses show short-term depression, the STN to SNr synapse should also show short-term depression to keep the SNr response small. Lindahl et al. (2013) further showed that short-term depression can have a big effect on the response of the BG when inputs last hundreds of milliseconds. Here, in our model we have only considered very short-lasting stimuli and therefore, short-term depression of synapses might not affect our results. In this work, so far, we have also ignored the effect of NMDA synapses. Such synapses can result in highly nonlinear synaptic integration (Du et al., 2017) and may affect the shape of the transient responses. The role of short-term dynamics and NMDA currents should be investigated in a more detailed model.

Dopamine has multiple effects on neuron’s excitability, synaptic strength, and synaptic plasticity (see Table 10). To better understand which one of these are most detrimental for the shape of the transient response, we individually perturbed six of the most crucial parameters (Fig. 6). This analysis revealed that the connection D2-SPN →GPe-TI is the most crucial for the shape of the transient response as it controls both LE and LI zones (Fig. 6). In addition, D1-SPN →SNr connection is expected to be crucial for determining the EI zone. We further corroborated these results by restoring the strength of D2-SPN →GPe-TI connection to their normal level while keeping all other parameters to their low-dopamine levels. This single change was effective in bringing the triphasic response in PD state closer to the one observed in healthy state.

**Figure 11.**
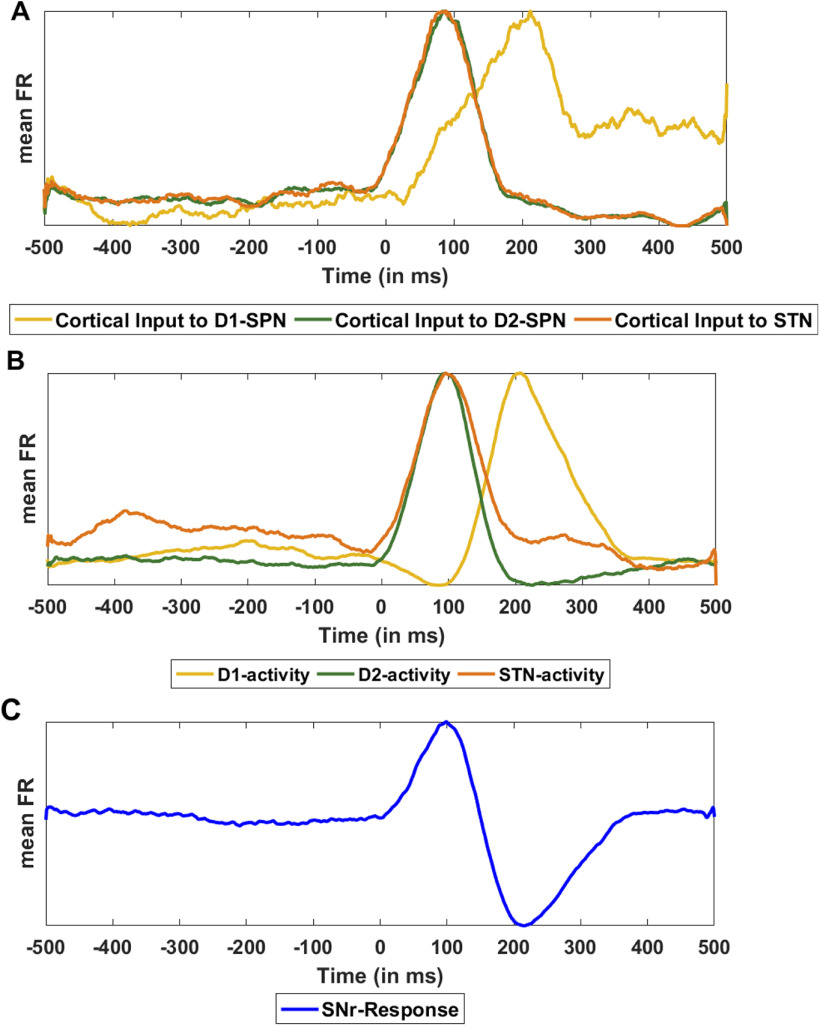
Schematic of putative cortical input profile to different BG neuron populations. ***A***, Schematic of cortical input to the D1-SPN, D2-SPN, and STN. ***B***, Neuronal activity in the D1-SPN, D2-SPN and STN in response to the cortical input shown in the panel ***A***. ***C***, Neuronal activity in the SNr in response to the cortical input shown in the panel ***A***.

Here, we have essentially characterized the impulse response of the BG network. The impulse response of the SNr does not resembled with the response observed in behavioral tasks (for example, see SNr response in Basso and Wurtz, 2002; Gulley et al. 2002; Wichmann and Kliem, 2004; and GPi response in Schwab et al., 2020). The temporal structure of the BG response means that in behavioral tasks inputs to the BG have a complex temporal structure. Our model can be used to predict the responses in different BG nuclei given a certain output pattern in the SNr or GPi. To illustrate this, we assumed a polyphasic response in activity of SNr. This particular response shape is inspired from the shape of the GPi response Schwab et al. (2020). In our model, such a polyphasic change in the GPi/SNr activity would require that STN and D2-SPNs change their activity before D1-SPNs (see Fig. 11). Moreover, the model gives information about the relative time scale of the transient activity in the D1-SPNs, D2-SPNs, and the STN neurons (see Fig. 11*B*) in response to the cortical input as shown in Figure 11*A*. Simultaneous measurement of activity in the striatum, STN and GPi/SNr could verify this prediction of the model. While deriving such input patterns, we only considered the three pathways. Other excitatory pathways like cortical innervation of the GPe could also affect the response. However, more experimental data are needed to infer the potential impact of such inputs.

In network models of β band oscillations when both STN and GPe are included, invariably STN ↔GPe connections emerge as a key parameter in shaping the oscillations (Holgado et al., 2010; Pavlides et al., 2015). In the full model of BG with both striatum and cortico-BG loop, STN ↔GPe may not be as important. Indeed Leblois et al. (2006) showed that altered interactions among direct and hyperdirect pathways are sufficient to induce oscillations. However, in the model by Leblois et al. (2006) GPe plays no role in generating oscillations – this is inconsistent with the experimental data (de la Crompe et al., 2020). In our model, consistent with the recent experimental data (de la Crompe et al., 2020) STN ↔GPe is not important for generating oscillations. In fact, in our model removal of STN ↔GPe-TI connections did not affect the oscillations (Fig. 9, blue bars). These observations combined with the experimental data (de la Crompe et al., 2020; Cruz et al., 2011) raise the question which network interactions generate oscillations, if not the STN-GPe loop. We have not explored this question in this work as the question requires a more systematic study. However, we speculate that besides the STN-GPe, the back-projections from GPe to striatum together with recurrent connections within the GPe can form an effective excitatory-inhibitory network necessary for generating oscillations. It is worth noting that previous experimental data (Mallet et al., 2006; de la Crompe et al., 2020; Sharott et al., 2017) and computational models (Kumar et al., 2011; Mirzaei et al., 2017; Bahuguna et al., 2020) provide a strong evidence that strengthening of D2-SPN →GPe-TI connection is also sufficient to induce β band oscillations/synchrony in the ongoing activity state of the BG. Thus, here, we provide a unified explanation of impaired transient response and ongoing activity in PD state. Our results highlight the importance of the GPe in controlling the dynamics and function of the BG.

In our model, emergence of β oscillations does not require synchrony among FSIs unlike some previous studies Damodaran et al. (2015). In our model, FSI synchrony could affect oscillations by modulating the D2-GPe-TI pathway and its effect could both strengthen or weaken the oscillations depending on the parameters. If synchronous firing of FSIs can reduce the firing rate of D2-SPNs, it will weaken the oscillations. If synchronized inhibition from FSIs can synchronize D2-SPNs then it can induce oscillations, regardless of the firing rate (Manferlotti et al., 2021).

Despite its simplicity, our model not only provides network interaction that shapes the properties of transient responses in the BG, but also clearly suggests that recurrent interactions within and between subnuclei of BG are crucial in shaping the transient response. We found that the duration of EE, EI, LI zones (and not LE zone) of the transient responses is largely robust to changes in the BG network interactions while the area/time of the different zones is not. This suggests that in *in vivo* data, we should find a narrow distribution of the duration of different zones and a wider distribution of the area/time of different zones. Next, our model predicts that by strengthening of cortical inputs, the normal shape of transient response cannot be restored in PD state. This prediction can be tested by either increasing the stimulus strength or by increasing the number of stimulated neurons (e.g., using optogenetic stimulation methods). Finally, the model predicts that by restoring the normal strength of D2-SPN →GPe-TI (or also by reducing the activity of D2-SPN), a near to healthy shape of transient response could be restored even in PD condition.

## Synthesis

Reviewing Editor: Alexxai Kravitz, Washington University in St. Louis Decisions are customarily a result of the Reviewing Editor and the peer reviewers coming together and discussing their recommendations until a consensus is reached. When revisions are invited, a fact-based synthesis statement explaining their decision and outlining what is needed to prepare a revision will be listed below. The following reviewer(s) agreed to reveal their identity: NONE.

Overall: Both reviewers were very positive about the manuscript and approach. They noted multiple strengths and only minor weaknesses that should be addressable within a 2-month timeframe. Essential comments that should be addressed in a revision:

1) Both reviewers brought up how the model simplifications were not justified, and some choices were not adequately clarified. Reviewer 1 noted a lack of D2-MSN modifications in the dopamine-depleted condition and lack of any short-term synaptic plasticity in their model. The impact of these simplifications should be discussed, and where possible tested with additional model simulations. Both reviewers also asked for more clarification on how levels of background excitatory inputs to each basal ganglia structure were chosen. Reviewer #1 also suggested running the full Lindahl and Kotaleski model for comparison, although I will leave it to the authors how to address this.

2) Reviewer 1 made a positive comment noting a potentially missed opportunity for an exciting addition to the manuscript: Using dopamine as a 0.0-1.0 gradient lends itself to some really interesting possibilities! In the paper, the PD condition is 0 dopamine, while the healthy condition is 0.8. Were other dopamine levels tested? Is there a specific threshold level at which the triphasic SNr response is disrupted, or is it a gradient with a smaller and smaller EI component as dopamine is reduced?

3) Reviewer 1 also asked for more information on FSIs in the model: FSI synchrony has previously been linked to beta-band oscillations in a striatal circuit model of dopamine depletion (Damodaran et al., 2015). The authors should comment on the level of synchrony of the FSIs in their model and whether this factor could contribute to the beta-band oscillations they observe. FSI synchrony could affect the D2-GPeTI output that they identify as critical for these oscillations.

4) Additional references should be included. Several previous experimental and computational works with direct relevance to this manuscript are not cited or discussed, but should be: Day et al. 2008, Lahiri and Bevan 2020, Cruz et al. 2011, Aristieta et al. 2020, Damodaran et al. 2015, Moran et al. 2011 PLOS CB.

Full reviews:

***********************

Reviewer 1:

***********************

The authors present a basal ganglia circuit model based off of the Lindahl and Kotaleski model. They use this model to test the specific neural connections necessary for the classical triphasic response seen in SNr neurons after cortical stimulation. Their dopamine depleted condition replicated the loss of the triphasic SNr response and they use sequential changes in the model to determine that this is predominately due to alterations in the indirect pathway MSNs connection with the prototypical GPe neurons. The most interesting predictions from this model are that 1. The pathological SNr response in dopamine depletion is due to ’;within basal ganglia circuitry’; rather that cortico-striatal connections. 2. The same circuit alterations that disrupt the triphasic SNr response also cause beta band oscillations traditionally seen in dopamine depleted animals. This paper is well written and presents interesting experimentally-testable predictions. However, I have the following concerns:

1. In line 237, the changes and simplifications relative to the Lindahl and Kotaleski model are described, but not justified. Why were these changes made? Do they affect the simulation outcome? This should be explained more thoroughly and perhaps a full model simulation should be run for comparison with the reduced model.

2. The authors do not modify their D2-MSNs for the dopamine-depleted condition, but there is evidence that D2-MSNs are modified in dopamine depletion conditions (Day et al., 2008) and these changes have been previously implemented in a striatal circuit model of dopamine depletion (Damodaran et al., 2015). Would the results be different if D2-MSN excitability was altered in the dopamine depletion condition?

3. In line 199, the authors state that ’;all neuronal populations received uncorrelated excitatory Poisson input spike-train, mimicking background inputs from cortex or thalamus.’; Does this include the GPe and SNr populations? The schematic in Figure 1 does not show cortical input to these structures. The GPe and SNr populations would not be expected to get direct excitatory input from the cortex or thalamus. Are there any studies recording mEPSCs or sEPSCs in GPe or SNr to justify this background excitatory input? And is there any justification for all neural populations receiving the same degree of background excitatory input? Example spike trains could be helpful here in understanding the overall effect of this background activity.

4. The authors do not implement any short-term synaptic plasticity in their model. However, synaptic adaptations such as short-term facilitation in striatonigral synapses and short-term depression in pallidonigral synapses are both well documented (Connelly et al., 2010) and are likely to affect circuit properties such as the development of beta-band oscillations.

5. The exponential adaptation of spiking in the SNr and GPe is interesting, showing example spike trains would be helpful here as well. Is the exponential adaptation of spiking critical the triphasic response in the SNr? Does the triphasic response depend on either the GPe or SNr being able to show rebound firing?

6. Using dopamine as a 0.0-1.0 gradient lends itself to some really interesting possibilities! In the paper, the PD condition is 0 dopamine, while the healthy condition is 0.8. Were other dopamine levels tested? Is there a specific threshold level at which the triphasic SNr response is disrupted, or is it a gradient with a smaller and smaller EI component as dopamine is reduced?

7. FSI synchrony has previously been linked to beta-band oscillations in a striatal circuit model of dopamine depletion (Damodaran et al., 2015). The authors should comment on the level of synchrony of the FSIs in their model and whether this factor could contribute to the beta-band oscillations they observe. FSI synchrony could affect the D2-GPeTI output that they identify as critical for these oscillations.

8. Several previous experimental and computational works with direct relevance to this manuscript are not cited or discussed, but should be: Day et al. 2008, Lahiri and Bevan 2020, Cruz et al. 2011, Aristieta et al. 2020, Damodaran et al. 2015, Moran et al. 2011 PLOS CB.

9. In Figure 7 the red and magenta colors are nearly indistinguishable, making it difficult to interpret the graphs.

***********************

Reviewer 2:

***********************

The authors of this manuscript seek to gain insight into how the BG network responds to transient cortical input after loss of dopamine, using, as a basis for exploring this, the previously established triphasic response in basal ganglia output typically elicited by cortical stimulation. They are building on previously well described network models. A further goal would appear to be to consider where one might intervene in the basal ganglia network to restore the output to a more normal pattern after loss of dopamine and thus compensate for the changes in network function associated with the parkinsonian state.

The authors present their methods, analysis, and results in a very clear, logical fashion. Modeling studies rely heavily on assumptions about the model’;s fidelity to in vivo studies and the authors explain their choices thoroughly. The assumptions the model rests upon, updates to the model and the various perturbations and alterations the authors conduct in the course of the study are explained in a readable manner that flows nicely from section to section. Their results indicate that differences in responses to stimulation of the cortex and STN are primarily due to alterations within the connectivity of BG circuits, rather than to cortico-BG projections. These changes also generated beta oscillations in the dopamine deprived network. D2 expressing striatal projection neurons (D2-SPN) projecting to prototypical (TI) GPE neurons, and STN projections to TI GPE neuronal projections proved most important in impacting the triphasic output. These projections also call attention to the role of the striatal projections to the GP in the emergence of beta oscillations in the parkinsonian state.

Overall, this is a very interesting and thoughtful study which provides insight into aspects of BG network physiology that can be further tested in animal models. The model leads to the prediction that restoring the strength of the D2 - SPN neurons projecting to the GPe-TI neurons should be effective in normalizing basal ganglia network function after loss of dopamine.

Comment s

1) Methods, page 8: lines 199 - 202 states: In our network model, all the neuronal populations received uncorrelated excitatory Poisson input spik-train, mimicking background inputs either from cortex or from thalamus. Each neuron in a give population received a different realization of Poisson type spikes with same parameters...tuned to ensure that the basal firing rates of different subnuclei were consistent with the in vivo recordings in rats.” It would be helpful if the text indicates whether the firing rate data used were from awake behaving, vs anesthetized states. It would also be helpful to either define the term ’;realization’; in the context of ’;realization of spikes’; or use a different term. Would ’;randomization’; be appropriate and more clear?

2) The discussion presents a good overview of the results in the context of the literature, and the constraints and limitations of the current version of the basal ganglia model.

3) The manuscript would benefit from some editing of the grammar in places.

## Author Response

Dear Dr. Kravitz

Thank you for giving us the opportunity to revise the manuscript. We would like to thank the two reviewers for their constructive feedback which has certainly improved the manuscript.

We have now prepared a revised version where all the changes are marked in crimson.

In the following we provide a point-by-point reply to the reviewers’; comments and our reply is marked in blue.

We look forward to hearing from you.

Best, Arvind on behalf of the co-authors

---------------------------------------------

Synthesis of Reviews:

Computational Neuroscience Model Code Accessibility Comments for Author (Required):

These authors will need to add a section on “Code Accessibility” containing the relevant info to comply with eNeuro’;s Policy on Computational Neuroscience Models. Currently they mention “Simulation code will be made available on github upon publication of the manuscript.” but this is not sufficient to comply.

We have now provided a link to the github repository from where the simulation code can be obtained.

Significance Statement Comments for Author (Required):

N/A

Comments on the Visual Abstract for Author (Required):

N/A

Synthesis Statement for Author (Required):

Overall: Both reviewers were very positive about the manuscript and approach. They noted multiple strengths and only minor weaknesses that should be addressable within a 2-month timeframe. Essential comments that should be addressed in a revision:

1) Both reviewers brought up how the model simplifications were not justified, and some choices were not adequately clarified. Reviewer 1 noted a lack of D2-SPN modifications in the dopamine-depleted condition and lack of any short-term synaptic plasticity in their model. The impact of these simplifications should be discussed, and where possible tested with additional model simulations. Both reviewers also asked for more clarification on how levels of background excitatory inputs to each basal ganglia structure were chosen. Reviewer #1 also suggested running the full Lindahl and Kotaleski model for comparison, although I will leave it to the authors how to address this.

To address this concern we have now performed new simulations where we explicitly modelled modulation of D2-SPNs and studied its impact on the transient response [140-143 in Methods and 424-430 in Results, Table 13]. In addition, we have discussed how short-term dynamics may affect our results. Essentially, we argue that transient response is too fast for short-term dynamics to manifest. Similarly, steady oscillations in the beta range are not likely to be affected by the short-term dynamics. [see Lines 272-276]

2) Reviewer 1 made a positive comment noting a potentially missed opportunity for an exciting addition to the manuscript: Using dopamine as a 0.0-1.0 gradient lends itself to some really interesting possibilities! In the paper, the PD condition is 0 dopamine, while the healthy condition is 0.8. Were other dopamine levels tested? Is there a specific threshold level at which the triphasic SNr response is disrupted, or is it a gradient with a smaller and smaller EI component as dopamine is reduced?

This is a very interesting suggestion. We have included a new figure in the revised version where we have tested the effect of varying dopamine levels in a continuous manner [See Figure 4]. For the triphasic response, both in terms of duration and area under the curve, change monotonically and smoothly as a function of dopamine modulation. However, when we consider the biphasic response, the EI phase appears only after dopamine level reaches 0.4. We added a new text to note this [see lines 430-436].

3) Reviewer 1 also asked for more information on FSIs in the model: FSI synchrony has previously been linked to beta-band oscillations in a striatal circuit model of dopamine depletion (Damodaran et al., 2015). The authors should comment on the level of synchrony of the FSIs in their model and whether this factor could contribute to the beta-band oscillations they observe. FSI synchrony could affect the D2-GPeTI output that they identify as critical for these oscillations.

In the steady state (both healthy and low-dopamine states) we did not include any synchrony in the FSI activity. While studying the transient response, the SPNs and FSIs were correlated as we activated a sizable fraction of neurons in a very short time. Whether FSI synchrony is linked to the emergence of oscillation is an interesting question. Computational models have shown that FSI synchrony is not crucial for the emergence of oscillations. Moreover, there is also no clear experimental data about the nature of synchrony in FSIs.

Whether FSI synchrony, if present, could affect the D2-GPe-TI pathway? Indeed, FSI synchrony can affect the indirect pathway in two ways: first it synchronous firing of FSIs can reduce the firing rate of D2 neurons and thereby weaken the oscillations. Second, synchronized inhibition from FSIs can in fact synchronize D2-SPNs. If D2-SPNs are firing in synchrony then that alone is sufficient to induce oscillations, irrespective of the firing rate (see Manferlotti et al. 2021). So if, indeed, FSI become synchronous they can amplify oscillations. However, to the best of our knowledge there is no evidence of FSI being synchronized in vivo. [See line 819-825]

4) Additional references should be included. Several previous experimental and computational works with direct relevance to this manuscript are not cited or discussed, but should be: Day et al. 2008, Lahiri and Bevan 2020, Cruz et al. 2011, Aristieta et al. 2020, Damodaran et al. 2015, Moran et al. 2011 PLOS CB.

We thank the reviewers for pointing us to the missed references. These references have been added in the appropriate places in the revised manuscript.

-

Full reviews:

***********************

Reviewer 1:

***********************

The authors present a basal ganglia circuit model based off of the Lindahl and Kotaleski model. They use this model to test the specific neural connections necessary for the classical triphasic response seen in SNr neurons after cortical stimulation. Their dopamine depleted condition replicated the loss of the triphasic SNr response and they use sequential changes in the model to determine that this is predominately due to alterations in the indirect pathway SPNs connection with the prototypical GPe neurons. The most interesting predictions from this model are that 1. The pathological SNr response in dopamine depletion is due to ’;within basal ganglia circuitry’; rather that cortico-striatal connections. 2. The same circuit alterations that disrupt the triphasic SNr response also cause beta band oscillations traditionally seen in dopamine depleted animals. This paper is well written and presents interesting experimentally-testable predictions. However, I have the following concerns:

1. In line 237, the changes and simplifications relative to the Lindahl and Kotaleski model are described, but not justified. Why were these changes made? Do they affect the simulation outcome? This should be explained more thoroughly and perhaps a full model simulation should be run for comparison with the reduced model.

The main focus of our work is to investigate how the structure of connectivity within and between basal ganglia subnetworks. Therefore, it was important to reduce model complexity e.g. removal of short-term dynamics, reduction of neurons in the striatum and change of the model of STN neurons. An added advantage of this was that we could simulate the model in a shorter time and therefore could do many more simulations to isolate the effect of many different connectivity parameters. Now we have explained this in the text [Lines 243-245 and 248-253].

We reduced the size of SPN populations from 75K to 4K. Lindahl and Kotaleski needed the larger size of striatal neurons as they were interested in action-selection and wanted to investigate the role of recurrent connections among D1- and D2-SPNs. It turns out that the same dynamics (low firing rate, sparse and uncorrelated activity) of SPNs can also be obtained in a smaller model with only 4K neurons. So, population size is not a key variable as long as the SPN activity remains asynchronous and sparse.

Next, we removed short-term dynamics from the synapse. As we have now explained in the revised manuscript removing the short-term dynamics of synapse does not affect our results [See lines 272-276].

Even though Lindahl and Kotaleski modelled STN as a two-dimensional model to mimic hyperpolarization induced bursts. In our model we do not force the STN neuron into such a state so there is no point in modelling these neurons as a two-dimensional model and LIF was sufficient. In fact, a number of previous studies have modelled STN neurons as LIF.

Finally, we would like to argue that it is not our aim to show that our reduced model is a faithful reproduction of the Lindahl and Kotaleski model. Each model is devised to address a specific question. Our model has sufficient complexity to reproduce the transient response and steady-state activity of the basal ganglia and to make testable predictions which are the only way to validate the model.

2. The authors do not modify their D2-SPNs for the dopamine-depleted condition, but there is evidence that D2-SPNs are modified in dopamine depletion conditions (Day et al., 2008) and these changes have been previously implemented in a striatal circuit model of dopamine depletion (Damodaran et al., 2015). Would the results be different if D2-SPNs excitability was altered in the dopamine depletion condition?

We thank the reviewer for pointing this out to us. We tested how including the effect of dopamine on D2-SPNs affects the transience response. We did not find any appreciable changes in the transient response. These results have been added in the new Table 13 and updated the text accordingly [140-143 in Methods and 424-430 in Results].

3. In line 199, the authors state that ’;all neuronal populations received uncorrelated excitatory Poisson input spike-train, mimicking background inputs from cortex or thalamus.’; Does this include the GPe and SNr populations? The schematic in Figure 1 does not show cortical input to these structures. The GPe and SNr populations would not be expected to get direct excitatory input from the cortex or thalamus. Are there any studies recording mEPSCs or sEPSCs in GPe or SNr to justify this background excitatory input? And is there any justification for all neural populations receiving the same degree of background excitatory input? Example spike trains could be helpful here in understanding the overall effect of this background activity.

In our model we drive the neurons with Poisson type spike trains. The purpose of this input is to ensure that the neuron firing rate is matched to the values measured in vivo. For, striatum this external input corresponds to the cortical and thalamic inputs. For the STN, this input corresponds to the cortical input as well as to the endogenous spontaneous activity of STN neurons. For the GPe and SNr this input ensures spontaneous activity of neurons. This activity may be endogenously generated or coming from presynaptic sources. In fact, recent data suggests that GPe neurons do get direct cortical inputs [Karube et al. 2019 eLife]. We have clarified this in the Methods section [Lines 203-207] and also modified Figure 1 accordingly.

4. The authors do not implement any short-term synaptic plasticity in their model. However, synaptic adaptations such as short-term facilitation in striatonigral synapses and short-term depression in pallidonigral synapses are both well documented (Connelly et al., 2010) and are likely to affect circuit properties such as the development of beta-band oscillations.

It is indeed possible that the temporal evolution of beta oscillations is affected by the short-term dynamics. And this becomes even more relevant when beta oscillations occur in short bursts. But here, at least in our current model, the focus is either on fast transient responses or steady-state power of beta oscillations. Because short-term dynamics affects the synapses only over a few spikes, it has no visible effect on the transient response. The steady-state effect of short-term dynamics is subject to the firing rate or oscillation frequency. The depression and facilitation time constants are typically of the order of 100ms. Therefore, the effects of short-term dynamics are strong only at high frequencies.

To demonstrate this we tested the transfer of oscillations across a synapse showing either short-term facilitation or short-term depression. For a range of parameters (consistent with the values used on Lindahl et al 2015) we found that short-term dynamics does not affect the transfer of beta band oscillations. Based on this observation we can argue that our model results about beta band oscillations will not be affected by short-term dynamics. However, short-term dynamics can induce counterintuitive results some time (e.g. see Tauffer and Kumar 2021) and in a future work the effect of short-term dynamics should be investigated more systematically especially in the case of beta bursts. We have also updated the Limitations of the model section [see Lines 272-276].

5. The exponential adaptation of spiking in the SNr and GPe is interesting, showing example spike trains would be helpful here as well. Is the exponential adaptation of spiking critical to the triphasic response in the SNr? Does the triphasic response depend on either the GPe or SNr being able to show rebound firing?

The ad-ex neuron model for SNr and GPe was retained from the Lindahl model. The adaptation of the neuron firing does not affect our results. We tested this by using LIF neurons for SNr population. Adding these results in the manuscript will only clutter the already long manuscript, therefore we have not included these results in the revised version of the manuscript.

We have also provided the raster plot of neuronal activities for GPe-TA, GPe-TI, STN and SNr based on the single trial data (from the 100 trials used to generate Figure 3) as an extended data Figure 3-1.

6. Using dopamine as a 0.0-1.0 gradient lends itself to some really interesting possibilities! In the paper, the PD condition is 0 dopamine, while the healthy condition is 0.8. Were other dopamine levels tested? Is there a specific threshold level at which the triphasic SNr response is disrupted, or is it a gradient with a smaller and smaller EI component as dopamine is reduced?

We thank the reviewer for raising this question. To address this question we have added a new figure in the manuscript [see Figure 4]. We found that the triphasic response, both in terms of duration and area under the curve, varies monotonically and smoothly as a function of dopamine level. However, when we consider the biphasic response, the EI phase appears only after dopamine level reaches 0.4. We added a new text to note this [see lines 430-436 ]. It is a different question where the transition from a triphasic response to biphasic response is continuous or discrete -- we have not addressed this specific question in our manuscript.

7. FSI synchrony has previously been linked to beta-band oscillations in a striatal circuit model of dopamine depletion (Damodaran et al., 2015). The authors should comment on the level of synchrony of the FSIs in their model and whether this factor could contribute to the beta-band oscillations they observe. FSI synchrony could affect the D2-GPeTI output that they identify as critical for these oscillations.

In our model there is no synchrony in the FSI population. However, when neurons are stimulated transient, FSI receive common input and they spike together.

Whether FSI synchrony, if present, could affect the D2-GPE-TI pathway. Indeed, FSI synchrony can affect the indirect pathway in two ways: first it synchronous firing of FSIs can reduce the firing rate of D2 neurons and thereby weaken the oscillations. Second, synchronized inhibition from FSIs can in fact synchronize D2-SPNs. If D2-SPNs are firing in synchrony then that alone is sufficient to induce oscillations, irrespective of the firing rate (see Manferlotti et al. 2021). So if, indeed, FSI become synchronous they can amplify oscillations. However, to the best of our knowledge there is no evidence of FSI being synchronized in vivo. We have also added a paragraph in the Discussion [see lines 819-825].

8. Several previous experimental and computational works with direct relevance to this manuscript are not cited or discussed, but should be: Day et al. 2008, Lahiri and Bevan 2020, Cruz et al. 2011, Aristieta et al. 2020, Damodaran et al. 2015, Moran et al. 2011 PLOS CB.

We have added the reference to Day et al. 2008, Damodaran et al. 2015, [see lines 428], Cruz et al. 2011 [see line 805] in the manuscript. However, we do not think that the following references are relevant to our work: Moran et al. 2011 PLOS CB, Aristieta et al. 2020, Lahiri and Bevan 2020.

9. In Figure 7 the red and magenta colors are nearly indistinguishable, making it difficult to interpret the graphs.

We have rendered the PD-biphasic in cyan and triphasic in red now. [see Figure 8].

***********************

Reviewer 2:

***********************

The authors of this manuscript seek to gain insight into how the BG network responds to transient cortical input after loss of dopamine, using, as a basis for exploring this, the previously established triphasic response in basal ganglia output typically elicited by cortical stimulation. They are building on previously well described network models. A further goal would appear to be to consider where one might intervene in the basal ganglia network to restore the output to a more normal pattern after loss of dopamine and thus compensate for the changes in network function associated with the parkinsonian state.

The authors present their methods, analysis, and results in a very clear, logical fashion. Modeling studies rely heavily on assumptions about the model’;s fidelity to in vivo studies and the authors explain their choices thoroughly. The assumptions the model rests upon, updates to the model and the various perturbations and alterations the authors conduct in the course of the study are explained in a readable manner that flows nicely from section to section. Their results indicate that differences in responses to stimulation of the cortex and STN are primarily due to alterations within the connectivity of BG circuits, rather than to cortico-BG projections. These changes also generated beta oscillations in the dopamine deprived network. D2 expressing striatal projection neurons (D2-SPN) projecting to prototypical (TI) GPE neurons, and STN projections to TI GPE neuronal projections proved most important in impacting the triphasic output. These projections also call attention to the role of the striatal projections to the GP in the emergence of beta oscillations in the parkinsonian state.

Overall, this is a very interesting and thoughtful study which provides insight into aspects of BG network physiology that can be further tested in animal models. The model leads to the prediction that restoring the strength of the D2 - SPN neurons projecting to the GPe-TI neurons should be effective in normalizing basal ganglia network function after loss of dopamine.

Comments

1) Methods, page 8: lines 199 - 202 states: In our network model, all the neuronal populations received uncorrelated excitatory Poisson input spik-train, mimicking background inputs either from cortex or from thalamus. Each neuron in a give population received a different realization of Poisson type spikes with same parameters...tuned to ensure that the basal firing rates of different subnuclei were consistent with the in vivo recordings in rats.” It would be helpful if the text indicates whether the firing rate data used were from awake behaving, vs anesthetized states. It would also be helpful to either define the term ’;realization’; in the context of ’;realization of spikes’; or use a different term. Would ’;randomization’; be appropriate and more clear?

We have revised the methods section accordingly. [Line 211]. The term ’;realization’; is a standard term to express a specific generation of random variables with some fixed parameters. So we do not see any confusion here.

2) The discussion presents a good overview of the results in the context of the literature, and the constraints and limitations of the current version of the basal ganglia model.

3) The manuscript would benefit from some editing of the grammar in places.

We thank the reviewer for pointing this out. We have now revised the manuscript with careful proofreading.

--
