# Erratum: Chakravarty et al., “Transient Response of Basal Ganglia Network in Healthy and Low-Dopamine State”

**DOI:** 10.1523/ENEURO.0572-24.2024

**Published:** 2025-01-17

In the article “Transient Response of Basal Ganglia Network in Healthy and Low-Dopamine State,” by Kingshuk Chakravarty, Sangheeta Roy, Aniruddha Sinha, Atsushi Nambu, Satomi Chiken, Jeanette Hellgren Kotaleski, and Arvind Kumar, which was published online on February 9, 2022, an affiliation for Arvind Kumar was accidentally omitted. The missing affiliation is SciLifeLab, Stockholm, Sweden. The corrected author affiliations are listed below.

Kingshuk Chakravarty,^1^ Sangheeta Roy,^1^ Aniruddha Sinha,^1^ Atsushi Nambu,^2,3^ Satomi Chiken,^2,3^ Jeanette Hellgren Kotaleski,^4,5^ and Arvind Kumar^4,6^

^1^TCS Research, Tata Consultancy Services, Kolkata 700160, India, ^2^Division of System Neurophysiology, National Institute for Physiological Sciences, Okazaki 444-8585, Japan, ^3^Department of Physiological Sciences, SOKENDAI (Graduate University for Advanced Studies), Okazaki 444-8585, Japan, ^4^Department of Computational Science and Technology, School of Computer Science and Communication, KTH Royal Institute of Technology, Stockholm SE-10044, Sweden, ^5^Department of Neuroscience, Karolinska Institute, Stockholm SE 171 77, Sweden, and ^6^SciLifeLab, Stockholm, Sweden

