# Supplementary figures and images for: Transient Response of Basal Ganglia Network in Healthy and Low-Dopamine State
Source: eNeuro. 2022 Mar 17;9(2):ENEURO.0376-21.2022. doi: 10.1523/ENEURO.0376-21.2022 (PMC8938981; doi:10.1523/ENEURO.0376-21.2022)

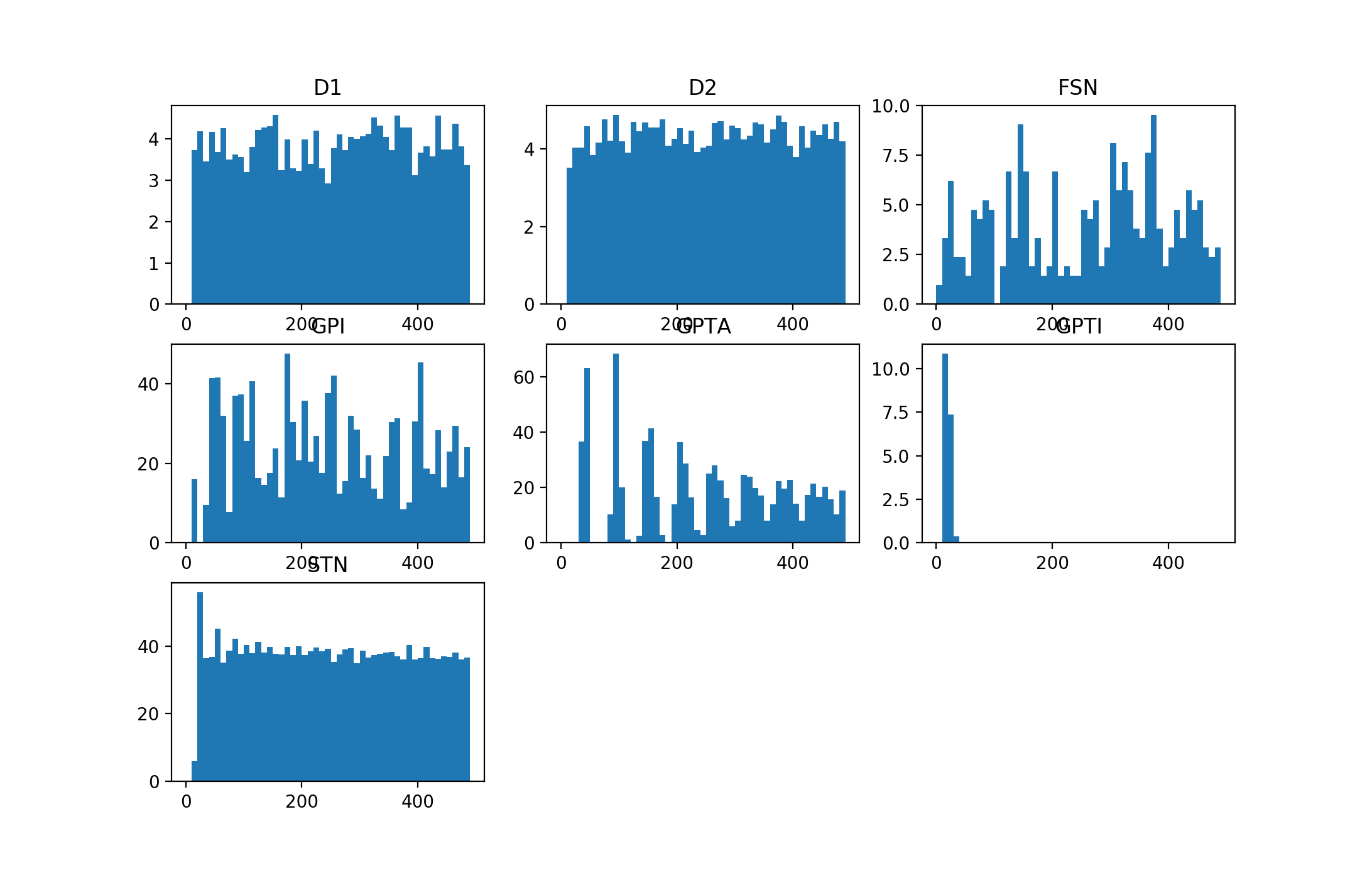

Supplement: Extended Data 1 — Basal ganglia transient response. Download Extended Data 1, ZIP file. [file enu-eN-NWR-0376-21-s03.zip › Basal-Ganglia-Transient-Response-main/BGcore/Simulations/BasalFiring/graphs/figure_1.png]

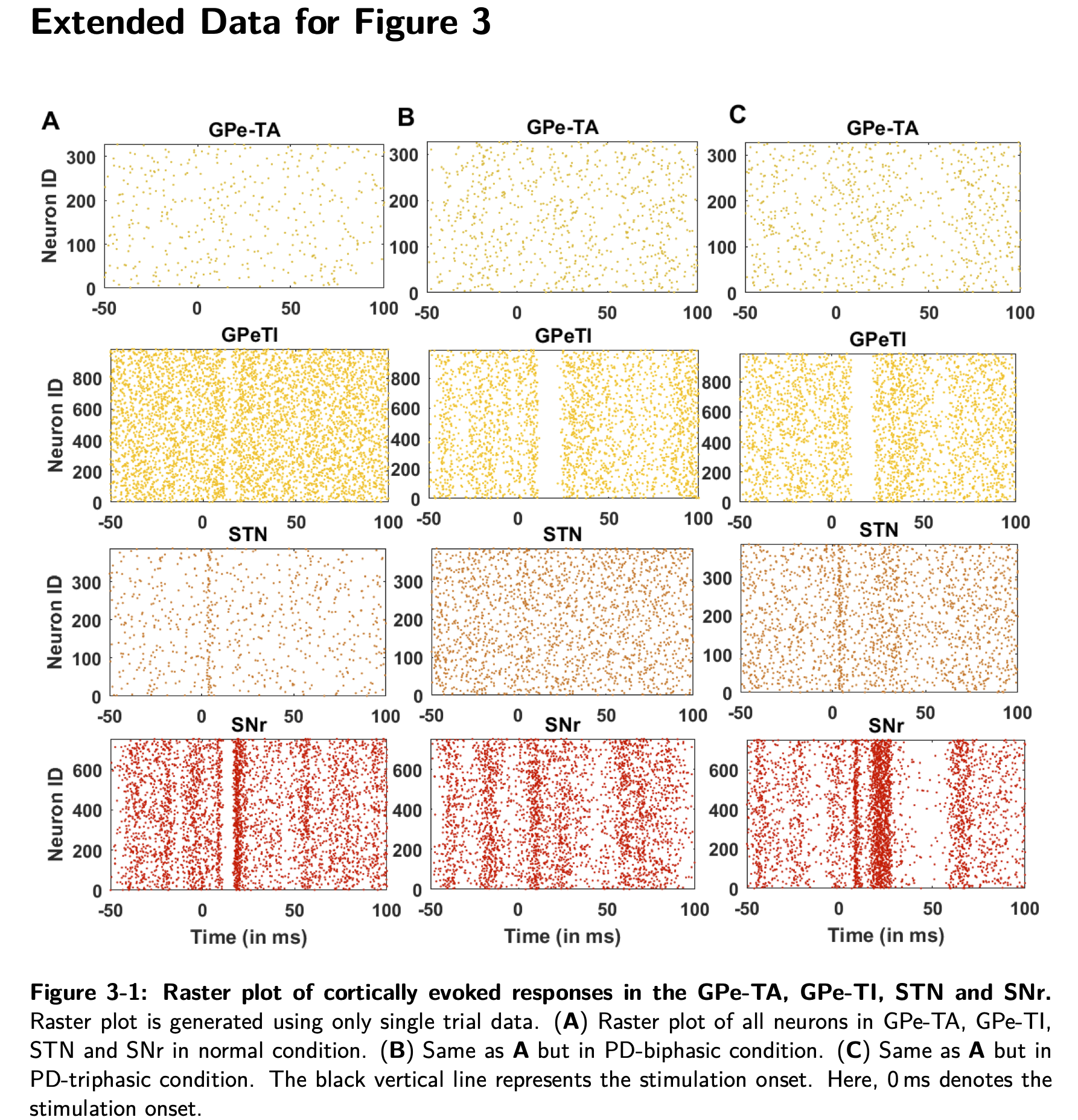

Supplement: Extended Data Figure 3-1 — Raster plot of cortically evoked responses in the GPe-TA, GPe-TI, STN, and SNr. Raster plot is generated using only single trial data. A, Raster plot of all neurons in GPe-TA, GPe-TI, STN, and SNr in normal condition. B, Same as A but in PD-biphasic condition. C, Same as A but in PD-triphasic condition. The black vertical line represents the stimulation onset. Here, 0 ms denotes the stimulation onset. Download Figure 3-1, TIF file. [file enu-eN-NWR-0376-21-s02.tif]
